# Supplementary material for: Addressing Inflammaging and Disease-Related Malnutrition: Adequacy of Oral Nutritional Supplements in Clinical Care
Source: Nutrients. 2024 Nov 29;16(23):4141. doi: 10.3390/nu16234141 (PMC11643944; doi:10.3390/nu16234141)
Supplement: Supplementary file 1 [file nutrients-16-04141-s001.zip › nutrients-3305957-supplementary.pdf]

Index

Supplementary Table S1: Classification of the analyzed FSMPs and supplements according to their macronutrients content, texture, flavour, osmolarity, and packaging

Supplementary Table S2: Classification of the analyzed FSMPs and supplements according to their micronutrients content

Supplementary Table S3: Cost analysis of the analyzed FSMPs and supplements

Supplementary Table S1. Classification of the analyzed FSMPs and supplements according to their macronutrients content, texture, flavour, osmolarity, and packaging. Abbreviations used: n.: number; Leu: leucine; Val: valine; Iso: isoleucine; An: animal; Pl: plant; EPA: eicosapentaenoic acid; DHA: docosahexaenoic acid; MCT: medium chain triglycerides; Osm: osmolarity; p: portion; b: brick; j: jar; s: sachet; FSMP: food for special medical purpose. Information was gathered from the companies' compedia and from their official websites.

**Product n. and type**	**Portion**	**Energy** (kcal/100ml, kcal/p, kcal/ml)	**Proteins and amino acids**							**Lipids**			**Carbohydrates**		**Glycemic Index**	**Texture and flavour**	**Osm** (mOsmol/l)	**Packaging** (component of the product, material, corresponding code)
			**Total** (g/100ml, g/p) **Added aa**	**Type**	**Leu** (g/p) **Ratio Leu: Val: Iso**	**Arg** (g/p) **Gln** (g/p)	**An source** (%)	**Pl source** (%)	**R An/Pl prot.**	**Total** (g/100ml; g/p)	**omega 3** (g/100 ml, g/p) **EPA+ DHA** (g/100 ml, g/p) (Ratio omega 6/omega3)	**MCT** (g/100 ml, g/p, % on total lipid)	**Total** (g/100m, g/p)	**Fiber** (g/100 ml, g/p); types of soluble fiber				
n.1 FSMP	40 g	376 (150.4/p) (3.76 kcal/g)	52 (20.8/p); 27 (11/p) EAA	whey protein, EAA	2.8/p<br>-	-<br>-	100%	-	-	3/p	0.15 (0.06/p) (R = 5.3)	-	9.5/p	3.2 (1.3/p); pectin, FOS	-	powder strawberry,vanilla	410 mOsmol/l	- jar e seal: FE 40 . metal<br>- cover: LDPE 4 . plastic<br>- measuring spoon: PP 5 . plastic
n.2 FSMP	200 ml	100 (200/b) (1 kcal/ml)	9.8 (19.6/b)	casein, whey protein	-<br>-	-<br>-	100%	-	-	2.1 (4.2/b)	0.09 (0.18/b) (R = 5)	-	10.3 (20.6/b)	0	-	liquid vanilla,chocolate,coffee	415 mOsmol/l	-jar: HDPE 2 plastic;<br>-top: HDPE 2 plastic;<br>-capsule: C/ALU 90 aluminium
n.3 FSMP	200 ml	151 (300/b)	10.4 (20.8/b);	whey protein, aa	3/b<br>-	-<br>-	100%	-	-	5.2 (10.4/b)	-	-	15.0 (30/b)	1.4 (2.8/b); pectin,	-	liquid berries,tropical vanilla	680 mOs	- brick: HDPE 2 . plastic;

		(1.51 kcal/ml)	0.72 (1.44/b) AA											FOS, GOS			mol/l	- top: HDPE 2 . plastic;<br>- capsule: C/ALU 90 . aluminium.
n.4 FSMP	200 ml	150 (300/b) (1.51 kcal/ml)	5.9 (11.8/b)	casein	- -	- -	100% (96.6% for chocolate)	- (3.4% for chocolate)	- (28 for chocolate)	5.8 (11.6/b)	0.27 (0.54/b) (R = 5)	-	18.4 (36.8/b)	0	-	liquid vanilla,banana,chocolate	455 mOsmol/l	- brick: HDPE 2 . plastic;<br>- top: HDPE 2 . plastic;<br>- capsule: C/ALU 90 . aluminium.
n.5 FSMP	200 ml	104 (208/b) (1.04 kcal/ml)	4.9 (9.8/b)	whey protein, soy protein	- -	- -	50%	50%	1	3.8 (7.6/b)	0.2 (0.4/b) (R = 4)	-	11.7 (23.4/b)	2 (4/b), of which 1.7 g (3.4/b) soluble; GOS	17-20	liquid vanilla,strawberry,cappuccino,chocolate	365 mOsmol/l	- brick: HDPE 2 . plastic;<br>- top: HDPE 2 . plastic;<br>- capsule: C/ALU 90 . aluminium.
n.6 FSMP	125 ml	240 (300/b) (2.4 kcal/ml)	9.6 (12/b)	casein, whey protein	- -	- -	100%	-	-	9.3 (11.6/b)	0.44 (0.55/b) (R = 5.07)	-	29.6 (37/b)	0	-	liquid vanilla,strawberry,coffee, banana, apricot,berries,chocolate,neutral	790 mOsmol/l	- brick: HDPE 2 . plastic;<br>- top: HDPE 2 . plastic;<br>- capsule: C/ALU 90 . aluminium.
n.7 FSMP	125 ml	240 (300/b) (2.4 kcal/ml)	9.6 (12/b)	casein, whey protein	- -	- -	100%	-	-	10.4 (13/b)	0.5 (0.62/b) (R = 5)	-	25.3 (31.6/b)	3.6 (4.5/b); FOS, GOS, pectin	-	liquid vanilla,coffee,strawberry	790 mOsmol/l	- brick: HDPE 2 . plastic;<br>- top: HDPE 2 . plastic;<br>- capsule: C/ALU 90 . aluminium.
n.8 FSMP	125 ml	245 (306.2/b)	9.6 (12/b)	casein, whey protein	- -	- -	100%	-	-	9.3 (11.6/b)	0.43 (0.54/b) (R = 5.3)	-	29.1 (36.4/b)	4 (5/b) of which 2.3 (2.9/b)	-	thick liquid vanilla,strawberry	495 mOsmol/l	- brick: HDPE 2 . plastic;<br>- top: HDPE 2 . plastic;

		(2.45 kcal/ml)												soluble; inulin, FOS, arabic gum, soy polysaccharides				- capsule: C/ALU 90 . aluminium.
n.9 FSMP	150 g	137 (205.5/j) (1.37 kcal/g)	7 (10.5/j)	whey protein	- -	- -	100%	-	-	4 (5/j)	0.182 (0.23/j) (R = 5.02)	-	17 (25.5/j)	2.6 (3.9/j) of which 1.9 (2.4/j) soluble; inulin, arabic gum, FOS, soy polysaccharides	-	pudding apple,strawberry	900 mOsmol/l	-
n.10 FSMP	125 ml	245 (306/b) (2.45 kcal/ml)	14.6 (18.25/b)	casein, whey protein	- -	- -	100%	-	-	9.6 (12/b)	0.5 (0.62/b) (R = 5.07)	-	25.1 (31.4/b)	0	-	liquid vanilla,strawberry,coffee,banana,peach-mango,berries	570 mOsmol/l	- brick: HDPE 2 . plastic;<br>- top: HDPE 2 . plastic;<br>- capsule: C/ALU 90 . aluminium.

n.11 FSMP	200 ml	150 (300/b) (1.5 kcal/ml)	9.6 (12/b)	protein isolate from pea and soybean	- -	- -	-	100%	-	5.8 (11.6/b)	0.29 (0.58/b) (R = 5.07)	-	18.6 (37.2/b)	0.05 of which 0 soluble	medium (-)	liquid coffee,mango-passion fruit	617 mOsmol/l	- brick: HDPE 2 . plastic;<br>- top: HDPE 2 . plastic;<br>- capsule: C/ALU 90 . aluminium.
n.12 FSMP	200 ml	400 (800/b) (4 kcal/ml)	5 (10/b)	casein, whey protein	- -	- -	100%	-	-	40.3 (80.6/b)	1.8 (3.6/b) (R = 5)	-	4.5 (9/b)	0	-	liquid neutral,strawberry	395 mOsmol/l	- brick: HDPE 2 . plastic;<br>- top: HDPE 2 . plastic;<br>- capsule: C/ALU 90 . aluminium.
n.13 FSMP	200 ml	124 (248/b) (1.24 kcal/ml)	9 (18/b)	casein, whey protein	- -	3/b -	100%	-	-	3.5 (7/b)	0.2 (0.4/b) (R = 5)	-	14.5 (29/b)	0	-	liquid vanilla,strawberry,chocolate	500 mOsmol/l	- brick: HDPE 2 . plastic;<br>- top: HDPE 2 . plastic;<br>- capsule: C/ALU 90 . aluminium.
n.14 FSMP	200 ml	150 (300/b) (1.5 kcal/ml)	10 (20/b)	milk protein	- -	- -	100%	-	-	6.7 (13.4/b)	-	-	12.1-12.4 (24.2-24.8/b)	0 (1/b for chocolate flavour); -	-	liquid vanilla,chocolate,strawberry	380-390 mOsmol/l	(plastic)
n.15 FSMP	200 ml	200 (400/b) (2 kcal/ml)	10 (20/b)	milk protein	2/b (1.8 : 1.3 : 1)	0.74/b 1.88/b	100%	-	-	7.8 (15.6/b)	0.27 (0.54/b) (R = 4.2)	-	22.5 (45/b)	0	-	liquid vanilla,berries,cappuccino,carrot	590-640 mOsmol	(plastic)
n.16 FSMP	125 g	200 (250/j)	10 (12.5/j)	milk protein	1.2/j	0.45/j 0.94/j	100%	-	-	7.8 (9,8/j)	0.25 (0.31/j)	-	22.5 (28.1/j); 22.4	0	-	cream strawberry,cappuccino	-	(plastic)

		(2 kcal/g)			(1.7 : 1.2 : 1)						(R = 4.32)		(28/j) for chocolate flavour					
n.17 FSMP	125 ml	320 (400/b) (3.2 kcal/ml)	16 (20/b)	milk protein	0.88/b (1.9 : 1.4 : 1)	1.3/b -	100%	-	-	16 (20/b)	1.5 (1.88/b) (R = 2.2)	-	28 (35/b)	0.4 (0.5/b) of which 0 soluble	-	thick liquid vanilla-caramel,hazelnut,mango,cappuccino	630-697-730-734 mOsmol/l	-
n.18 FSMP	120 ml	500 (600/b) (5 kcal/ml)	0	-	-	-	-	-	-	53.8 (64.6/b)	3.6 (4.4/b) (R = 2.3)	13.9 (16.7/b) (25.8%)	4 (4.8/b)	0.4 (0.48/b); -	-	liquid neutral	-	-
n.19 FSMP	200 ml/b	150 (300/b) (1.5 kcal/ml)	7.5 (15/b)	milk protein	1.5/b (1.7 : 1.2 : 1)	0.56/b 1.14/b	100%	-	-	7 (14/b)	0.45 (0.9/b) EPA + DHA = 0.054 (0.11/b) (R = 2.27)	1.2 (2.4/b) (17%)	13.1 (26.2/b)	2 (4/b), soluble; tapioca dextrins	low (-)	liquid vanilla,cappuccino	350-360-390 mOsmol/l	-
n.20 FSMP	200 ml	240 (480/b) (2.4 kcal/ml)	14.4 (28.8/b)	whey protein, casein	2.8/b -	- -	100%	-	-	9.4 (18.8/b)	0.65 (1.3/b) (R = 2.5)	-	24.4 (48.8/b)	0	-	liquid hazelnut,vanilla,apricot-peach,cappuccino	570-630-660 mOsmol/l	-

n.21 FSMP	125 ml	240 (300/b) (2.4 kcal/ml)	14.4 (18/b)	whey protein, casein	1.75/b<br>-	-<br>-	100%	-	-	9.4 (18.8/b)	0.65 (1.3/b) (R = 2.5)	-	24 (30/b)	0	-	liquid vanilla	630 mOsmol/l	-
n.22 FSMP	200 ml	150 (300/b) (1.5 kcal/ml)	6 (12/b)	casein, whey protein, soy protein	-<br>-	-<br>-	-	-	-	5 (10/b)	0.38 (0.76/b) EPA+ DHA = 0.05 (0.1/b) (R = 3.17-3.44)	0.65 - 0.76 (1.3 - 1.5/b) (13-15 %)	20 (40/b)	< 0.1 (< 0.2/b) 0.6 (1.2/b) for chocolate flavour	-	liquid banana,chocolate,strawberry,vanilla	455-505 mOsmol/l	(plastic)
n.23 FSMP	200 ml	150 (300/b) (1.5 kcal/ml)	6 (12/b)	casein, soy protein	-<br>-	-<br>-	-	-	-	5 (10/b)	0.39-0.43 (0.78-0.86/b) EPA+ DHA = 0.21 (0.42/b) (R = 1.05-1.08)	2.3-2.5 (4.6 - 5/b) (46-50 %)	19 (38/b)	2 (4/b) of which 1.4 (2.8/b) soluble; inulin, dextrins	-	liquid vegetable,chicken curry	425-440 mOsmol/l	(plastic)
n.24 FSMP	200 ml	103 (206/b) (1.03 kcal/ml)	4.1 (8.2/b)	casein, soy protein	-<br>-	-<br>-	-	-	-	3.5 (7/b)	0.3 (0.6/b) EPA+ DHA = 0.18 (0.36/b)	-	12 (24/b)	2.1 (4.2/b) of which 1.5 (3/b) soluble;	-	liquid vanilla	220 mOsmol/l	(plastic)

											(R = 1.17)			inulin, dextrins				
n.25 FSMP	200 ml	200 (400/b) (2 kcal/ml)	9 (18/b)	casein, whey protein	- -	- -	100%	-	-	8 (16/b)	0.62-0.63 (1.24-1.26/b) EPA+ DHA = 0.08 (0.16/b) (R = 2.97-3.03)	1.2-1.9 (2.4-3.8/b) (15 %)	21.4 (42.8/b)	2.5 (5/b), soluble; inulin, dextrins	-	liquid caramel,chocolate praline,cherry	590-620 mOsmol/l	(plastic)
n.26 FSMP	200 ml	150 (300/b) (1.5 kcal/ml)	10 (20/b)	casein, whey protein, soy protein	- -	- -	-	-	-	5.5 (11/b)	0.43 (0.86/b) EPA+ DHA = 0.05 (0.1/b) (R = 3.18)	0.83-0.85 (1.66-1.7/b) (15 %)	15 (30/b)	0.1 (0.2/b) 0.32 for chocolate flavour (0.64/b)	-	liquid chocolate praline,ananas-coconut,vanilla	460-485 mOsmol/l	(plastic)
n.27 FSMP	200 ml	108 (216/b) (1.08 kcal/ml)	4.8 (9.6/b)	whey protein	- -	- -	100%	-	-	5.6 (11.2/b)	1.2 (2.4/b) EPA+ DHA = 1 (2/b) (R = 0.25)	-	9.2 (18.4/b)	1 (2/b), soluble; pectin	low (21)	liquid raspberry,peach	698 mOsmol/l	-
n.28 FSMP	200 ml	180 (360/b)	10 (20/b)	milk protein	- -	- -	100%	-	-	7.4 (14.7/b)	n.a	-	18.5 (37/b)	0; 0.5 (1/b)	-	liquid peach-apricot,vanill	500-550 mOs	(plastic brick and external packaging paper)

		(1.8 kcal/ml)												for chocolate flavour		a,chocolate,coffee,red fruits,neutral	mol/l	
n.29 FSMP	200 ml	200 (400/b) (2 kcal/ml)	10 (20/b)	milk protein	- -	- -	100%	-	-	8 (16/b)	-	-	22 (44/b); 21.8 (43.6/b) for chocolate version	0; 0.5 (1/b) for chocolate flavour	-	liquid vanilla,chocolate,coffee,peach-apricot	480-490 mOsmol/l	(plastic jar and external packaging paper)
n.30 FSMP	200 ml	200 (400/b) (2 kcal/ml)	10 (20/b)	milk protein	- -	- -	100%	-	-	7.4 (14.8/b)	-	-	22 (44/b)	3 (6/b), soluble; inulin	-	liquid cookie,strawberry,caramel	600-625 mOsmol/l	(plastic jar and external packaging paper)
n.31 FSMP	200 ml	150 (300/b) (1.5 kcal/ml)	10 (20/b)	milk protein	- -	- -	100%	-	-	5.5 (11/b)	0.5 (0.9/b) (R = 2.33)	-	13.4 (26.8/b)	3.5 (7/b) of which 0 soluble	low (20)	liquid vanilla,coffee	250 mOsmol/l	(plastic jar and external packaging paper)
n.32 FSMP	200 ml	131 (261/b) (1.31 kcal/ml)	4.1 (8.2/b)	soluble milk protein	- -	- -	100%	-	-	0	-	-	26 (52/b)	5.1 (10.2/b) of which 0 soluble	-	liquid orange	800 mOsmol/l	(plastic jar and external packaging paper)
n.33 FSMP	125 g	180 (225/j)	10 (12.5/j)	whey protein	- -	- -	100%	-	-	8 (10/j)	-	-	16-16.8 (20-21/j)	0.6 (0.75/j)	-	cream	-	(plastic little jar, aluminium top

		(1.8 kcal/g)												; 2 (2.5/j) for chocolate flavour		vanilla,chocolate,praline,apricot		and external packaging paper)
n.34 FSMP	125 g	151 (188.8/j) (1.51 kcal/g)	10 (12.5/j)	milk protein	- -	- -	100%	-	-	5 (6.3/j)	0.6 (0.75/j) ; 0.2 (0.3/j) for chocolate flavour (R = 0.5)	-	16.5 (20.6/j) ; 16.4 (20.5/j) for apricot flavour	0-0.6 (0-0.75/j)	-	cream vanilla,coffee,caramel,apricot,chocolate	-	(plastic little jar, aluminium or plastic top and external packaging paper)
n.35 FSMP	125 g	180 (225/j) (1.8 kcal/g)	10.4 (13/j); 10 (12.5/j) for vanilla flavour	isolated milk protein	- -	- -	100%	-	-	9 (11.3/j)	-	-	12.4 (15.5/j) 13.2 (16.4/j) for vanilla	4 (5/j); 3,2 (4/j) for vanilla flavour of which 0 soluble	-	cream cocoa,vanilla	-	(plastic little jar, aluminium or plastic top and external packaging paper)
n.36 FSMP	237 ml	144 (341/b) (1.44 kcal/ml)	7.6 (18/b)	whey protein, casein	- -	4.3/b -	100%	-	-	3.9 (9.2/b)	0.6 (1.42/b) EPA + DHA =	1.1 (2.6/b) (28 %)	18.9 (44.7/b)	1.4 (3.3/b), soluble; PHGG	-	liquid vanilla,coffee,tropical	680 mOsmol/l	- brick paper C/PAP84 . recycle - external coating paper PAP21 . recycle

											0.5 (1.2/b) (R = 0.9)							
n.37 FSMP	200 ml	100 (200/b) (1 kcal/ml)	4 (8/b)	whey protein	-<br>-	-<br>-	100%	-	-	3.7 (7.4/b)	0.035 (0.07/b) (R = 8.49)	2.5 (5/b) (68 %)	12.7 (25.4/b)	0	-	liquid vanilla	280 mOsmol/l	-brick plastic HDPE 2 . recycle<br>-top plastic HDPE 2 . recycle<br>-film inside aluminium C/ALU 90 . recycle<br>-external packaging paper/cardboard PAP 21 . recycle
n.38 FSMP	125 ml	205 (256/b) (2.05 kcal/ml)	9.2 (11.5/b)	whey protein, casein	-<br>-	-<br>-	100%	-	-	8.7 (11/b)	1.4 (1.75/b) EPA+DHA = 1.4 g (1.75/b) (R = 3.8)	-	21 (26/b)	2.6 (3.2/b), soluble; FOS, GOS	-	liquid neutral,peach-vanilla,mango-peach	630 mOsmol/l	-brick plastic HDPE 2 . recycle<br>-top plastic HDPE 2 . recycle<br>-film inside aluminium C/ALU 90 . recycle<br>-external packaging paper/cardboard PAP 21 . recycle
n.39 FSMP	200 ml	160 (320/b) (1.6 kcal/ml)	10 (20/b); 4.6 (9.3/b) EAA	whey protein, casein, EAA	-<br>-	-<br>-	100%	-	-	7.8 (14.8/b)	0.35 (0.7/b) EPA+DHA = 0.165 (0.33/b) (R = 2)	1.2 (2.4/b) (16 %)	12.6 (25.2/b)	1.5 (3.0/b), soluble; FOS, inulin	-	liquid vanilla-tropical,caramel-banana	-	-brick plastic HDPE 2 . recycle<br>-top plastic HDPE 2 . recycle<br>-film inside aluminium C/ALU 90 . recycle<br>-external packaging

																		paper/cardboard PAP 21 . recycle
n.40 FSMP	200 ml	150 (300/b) (1.5 kcal/ml)	5 (10/b)	whey protein, casein	- -	- -	100%	-	-	0	-	-	32.5 (65/b)	0	-	liquid peach tea with mint	615 mOsmol/l	-brick plastic HDPE 2 . recycle -top plastic HDPE 2 . recycle -film inside aluminium C/ALU 90 . recycle -external packaging paper/cardboard PAP 21 . recycle
n.41 FSMP	125 ml	225 (281/b) (2.25 kcal/ml)	14 (17.5/b)	whey protein, casein	1.9/b -	- -	100%	-	-	8.7 (11/b)	0.57 (0.71/b) (R = 2.8)	-	22.6 (28/b)	0	-	liquid vanilla,coffee ,caramel	620 mOsmol/l	-brick plastic HDPE 2 . recycle -top plastic HDPE 2 . recycle -film inside aluminium C/ALU 90 . recycle -external packaging paper/cardboard PAP 21 . recycle
n.42 FSMP	200 ml	151 (302/b) (1.51 kcal/ml)	5.6 (11.2/b)	whey protein, casein	- -	- -	100%	-	-	5 (10/b)	- (R = 7.5)	-	21 (42/b)	0	-	liquid vanilla,apricot,chocolate,strawberry-raspberry	488 mOsmol/l	-brick plastic HDPE 2 . recycle -top plastic HDPE 2 . recycle -film inside aluminium C/ALU 90 . recycle -external packaging paper/cardboard PAP 21 . recycle

n.43 FSMP	200 ml	160 (320/b) (1.6 kcal/ml)	10 (20/b)	whey protein, casein	- -	- -	100%	-	-	6.2 (12.4/b)	- (R = 2.7)	-	16 (32/b)	0	-	liquid vanilla,chocolate,strawberry	480-550 mOsmol/l	-brick plastic HDPE 2 . recycle -top plastic HDPE 2 . recycle -film inside aluminium C/ALU 90 . recycle -external packaging paper/cardboard PAP 21 . recycle
n.44 FSMP	200 ml	200 (400/b) (2 kcal/ml)	9 (18/b)	whey protein, casein	- -	- -	100%	-	-	8.7 (17.4/b)	- (R = 2.7)	-	20 (40/b)	2.5 (5/b), soluble; FOS, GOS	-	liquid vanilla,coffee,neutral, strawberry	520 mOsmol/l	-brick plastic HDPE 2 . recycle -top plastic HDPE 2 . recycle -film inside aluminium C/ALU 90 . recycle -external packaging paper/cardboard PAP 21 . recycle
n.45 FSMP	200 ml	160 (320/b) (1.6 kcal/ml)	9 (18/b)	whey protein, casein	- -	- -	100%	-	-	6.3 (12.6/b)	- (R = 2.71)	-	15.7 (31.4/b)	2.5 (5/b), soluble; PHGG, acacia gum, FOS, inulin	low (30)	liquid vanilla,strawberry	300 mOsmol/l	-brick plastic HDPE 2 . recycle -top plastic HDPE 2 . recycle film inside aluminium C/ALU 90 . recycle -external packaging paper/cardboard PAP 21 . recycle
n.46	200 ml	127 (254/b)	9 (18/b)	whey prote	1.7/b	1/b -	100%	-	-	3 (6/b)	-	-	16 (32/b)	0	-	liquid vanilla,coffee	466 mOs	-brick plastic HDPE 2 . recycle

FSMP		(1.27 kcal/ml)		in, casein	(1.9 : 1.2 : 1)						(R = 2.7)						mol/l	-top plastic HDPE 2 . recycle film inside aluminium C/ALU 90 . recycle -external packaging paper/cardboard PAP 21 . recycle
n.47 FSMP	200 ml	125 (250/b) (1.25 kcal/ml)	9.4 (18.8/b)	milk protein	- -	- -	100%	-	-	3.5 (7/b)	< 0.5 (<1/b)	-	14 (28/b)	0	-	liquid apricot,chocolate,strawberry,vanilla	429 mOsmol/l	-jar paper/cardboard C/PAP 85 . recycle -cover plastic P 5 . recycle - film inside aluminium ALU 41 . recycle
n.48 supplement	30 g	357 (107/p) (3.57 kcal/g)	31 (9.3/p)	milk protein	- -	- -	100%	-	-	1 (0.3/p)	-	-	56 (16.7/p)	0	-	powder vanilla,chocolate,coffe, neutral	-	-brick plastic HDPE 2 . recycle -top plastic HDPE 2 . recycle -film inside aluminium C/ALU 90 . recycle
n.49 fortified food	125 g	170 (212.5/j) (1.7 kcal/g)	9.5 (11.9/j)	whey protein, casein	- -	- -	100%	-	-	7.3 (9.1/j)	-	-	16 (20/j)	1 (1.25/j), soluble; guar gum	-	pudding vanilla,chocolate,hazelnut	-	small jar plastic 7 . recycle film inside aluminium C/ALU 90 . recycle external packaging paper/cardboard PAP 21 . recycle
n.50 FSMP	200 ml	150 (300/b)	7 (14/b)	whey protein	- -	- -	100%	-	-	0	-	-	30.5 (61/b)	0	-	liquid orange	725 mOs	brick plastic HDPE 2 . recycle

		(1.5 kcal/b)															mol/l	top plastic HDPE 2 . recycle<br>film inside aluminium C/ALU 90 . recycle
n.51 fortified food	100 g	71 (71/j) (0.71 kcal/g)	0.9 (0.9/j)	milk cream	-<br>-	-<br>-	100%	-	-	1.8/j	-	-	12/j	2 (2/j), soluble; ?	-	pudding apple-vanilla	-	small jar plastic 7 . recycle<br>closing film plastic C/LDPE 90 . recycle<br>external packaging paper/cardboard PAP 21 . recycle
n.52 FSMP	200 ml	100 (200/b) (1 kcal/ml)	7 (14/b)	milk protein	-<br>-	-<br>-	100%	-	-	2.7 (5.4/b)	-	-	10.9 (21.8/b)	2 (4/b), soluble; PHGG, acacia gum, FOS, inulin	low (43)	liquid vanilla,strawberry	218 mOsmol/l	brick plastic HDPE 2 . recycle<br>top plastic HDPE 2 . recycle<br>film inside aluminium C/ALU 90 . recycle
n.53 FSMP	125 g	142 (177.5/j) (1.42 kcal/g)	8.8 (11/j)	whey protein, casein	-<br>-	-<br>-	100%	-	-	4.7 (5.9/j)	-	-	14 (18/j)	3.4 (4.2/j), soluble; guar gum, inulin	low (43)	cream vanilla,coffee	-	small jar plastic 7 . recycle<br>film inside aluminium C/ALU 90 . recycle<br>external packaging paper/cardboard PAP 21 . recycle
n.54 FSMP	200 ml	204 (408/b) (2.04 kcal/ml)	10 (20/b)	milk protein	-<br>-	-<br>-	100%	-	-	8 (16/b)	-	-	23 (46/b)	0.1 (0.2/b)	-	liquid apricot,coffee,vanilla,caramel,chocolate,red fruits	367-440 mOsmol/l	(jar plastic and external packaging paper)

n.55 FSMP	200 ml	204 (408/b) (2.04 kcal/ml)	10 (20/b)	milk protein	- -	- -	100%	-	-	8.1 (16.2/b)	-	-	21.5 (43/b)	2.5 (5/b), soluble; FOS	-	liquid coffee,strawberry,vanilla	364-404 mOsmol/l	-
n.56 FSMP	200 ml	151 (302/b) (1.51 kcal/ml)	6.1 (13.2/b)	milk protein	- -	- -	100%	-	-	6.1 (12.2/b)	-	-	17.9 (35.8/b)	0	-	liquid cocoa,red fruits,vanilla	344-401 mOsmol/l	-
n.57 FSMP	200 ml	150 (300/b) (1.5 kcal/ml)	5 (10/b)	milk protein	- -	- -	100%	-	-	0	-	-	32.5 (65/b)	0	-	liquid orange,berries,apple	720 mOsmol/l	-
n.58 FSMP	200 ml	150 (300/b) (1.5 kcal/ml)	10 (20/b)	milk protein	- -	- -	100%	-	-	6.8 (13.6/b)	-	-	11 (22/b)	2.5 (5/b)	low (-)	liquid coffee,red fruits,vanilla	213-263 mOsmol/l	-
n.59 FSMP	200 ml	100 (200/b) (1 kcal/ml)	5.7 (11.4/b)	milk protein, pea protein, L-carnitine, L-taurine	- -	- -	-	-	-	4.4 (8.8/b)	0.81 (1.62/b) EPA + DHA = 0.0675 (0.135/b) (R = 0.15)	-	8.4 (16.8/b)	1.8 (3.6/b) of which 1.44 (2.88/b) soluble; inulin	-	liquid cocoa,vanilla	345 mOsmol/l	-

n.60 FSMP	200 ml	125 (250/b) (1.25 kcal/ml)	6.3 (12.6/b)	milk protein, pea protein, L-carnitine, L-taurine	- -	- -	-	-	-	4.9 (/b)	0.13 (0.26/b) EPA + DHA = 0.338 (0.676/b) (R = 7)	-	13.1 (26.2/b)	1.7 (3.4/b) of which 1.4 (2.8/b) soluble; FOS	-	liquid vanilla	303 mOsmol/l	-
n.61 FSMP	200 ml	151 (302/b) (1.51 kcal/ml)	8.3 (16.5/b)	milk protein, pea protein,-L-carnitine, L-taurine	- -	2.1/b -	-	-	-	5 (10/b)	0.77 (1.54/b) EPA + DHA = 0.399 (0.798/b) (R = 1.04)	-	17.3 (34.6/b)	1.7 (3.4/b) of which 1.4 (2.8/b) soluble; FOS	-	liquid vanilla,tropical	366 mOsmol/l	-
n.62 FSMP	125 g	150-163 (187.5-203.8/j) (1.5-1.56 kcal/g)	10 (12.5/j)	milk protein	- -	- -	100%	-	-	5-6.1 (6.3-7.6/j)	-	-	14.8-15.9 (18.5-19.9/j)	1.3-2.2 (1.6-2.8/j) of which 0 soluble	-	cream apricot,coffee,caramel,chocolate,lemon, vanilla	-	-
n.63 FSMP	125 g	203 (253.7/j)	10.8 (13.5/j)	milk protein	- -	- -	100%	-	-	9.7 (12.1/j)	-	-	18 (22.5/j)	< 0.5/j	-	cream apricot-peach,coffee, caramel,choc	-	-

		(2.03 kcal/g)														olate,lemon,vanilla		
n.64 fortified food	125 g	153-156 (191.2-195/j) (1.53-1.56 kcal/g)	7.2-7.4 (9-9.2/j)	milk protein	- -	- -	100%	-	-	4.6-5.5 (5.8-6.9/j)	-	-	18.1-20.7 (22.6-25.9/j)	0.9-1.2 (1.1-1.5/j), soluble; ?	-	puree apple,apple-banana,apple-plum	-	-
n.65 FSMP	125 g	150 (187/j) (1.5 kcal/g)	10 (12.5/j)	milk protein	- -	- -	100%	-	-	6.1 (7.6/j)	-	-	12.2 (15.3/j)	3 (3.8/j) including soluble fiber; inulin	low (13.4-14.3)	cream apricot,coffee,lemon,vanilla	482-513 mOsmol/l	-
n.66 fortified food	125 g	160-162 (201-202/j) (1.6-1.62 kcal/g)	11.6-12.6 (14.5-15.8/j)	milk protein	- -	1.8-1.9/j -	100%	-	-	5.6-5.9 (7-7.4/j)	-	-	14.5-15.9-16.2 (18.1-19.9-20.3/j)	0	-	cream coffee,chocolate, vanilla-caramel	-	-
n.67 FSMP	200 ml	150 (300/b) (1.5 kcal/ml)	6.25 (12.5/b)	Na caseinate, whey protein isolate, soy protein	1.1/b (1.9 : 1.2 : 1)	0.48/b 1.26/b	87.5%	12.5%	7	4.92 (9.84/b)	0.19 (0.38/b) (R = 5.7)	-	20.2 (40.4/b)	0	-	liquid chocolate,banana,strawberry,berries,vanilla	517 mOsmol/l	Brick C/PAP 84 paper Box PAP 20 paper Closing film PP5 plastic

				isolate														
n.68 FSMP	125 g	137 (171.2/j) (1.37 kcal/g)	5.7 (7.1/j)	isolated whey protein, isolated soy protein	0.68/j (2 : 1.2 : 1)	0.25/j -	87.5%	12.5%	7	4.47 (5.59/j)	0.165 (0.21/j) (R = 6)	-	18.4 (23/j)	0	-	cream chocolate,banana,vanilla	602 mOsmol/l	Cup PP5 plastic Closing film C/ALU 90 Aluminum External box PAP 21 paper
n.69 FSMP	220 ml	150 (330/b) (1.5 kcal/ml)	9.1 (20/b)	Na caseinate, isolated and concentrated whey protein, isolated soy protein	1.78/b (1.8 : 1.15 : 1) β-HMB = 1.21 g/b	0.77/b 1.98/b	85%	15%	17 per brick anim / 3 per brick plant = 5.67	4.8 (10.6/b)	0.26 (0.57/b) (R = 5.6)	-	16.8 (37/b)	0.75 (1.65/b), soluble; FOS	-	liquid chocolate,vanilla,coffee,banana,strawberry	557 mOsmol/l	brick HDPE 2 plastic Top PP 5 plastic Label PETG 7 plastic Closing film C/ALU 90 aluminum External box PAP 21 paper
n.70 FSMP	200 ml	200 (400/b) (2 kcal/ml)	8.4 (16.8/b)	isolate whey protein, Na	1.5/b (1.8 : 1.2 : 1)	0.56/b 1.82/b	100%	-	-	8.9 (17.2/b)	0.21 (0.42/b) (R = 9.2)	-	21 (42/b)	1 (2/b), soluble; FOS	-	liquid vanilla	527 mOsmol/l	brick HDPE 2 plastic Top HDPE 2 plastic Label PETG 7 plastic

				casei nate														Closing film C/ALU 90 aluminum External box PAP 21 paper
n.71 FSMP	125 ml	240 (300/b) (2.4 kcal/ml)	10.2 (12.75/b)	concentrated and/or isolated whey protein, hydrolyzed Na caseinate	1.12/b (1.8 : 1.3 : 1)	0.7/b -	100%	-	-	9.35 (11.7/b)	0.34 (0.42/b) (R = 6.1)	-	28.8 (36/b)	0	-	liquid strawberry,vanilla,banana,coffee	675 mOsmol/l	brick HDPE 2 plastic Top HDPE 2 plastic Label PETG 7 plastic Closing film C/ALU 90 aluminum External box PAP 21 paper
n.72 FSMP	200 ml	150 (300/b) (1.5 kcal/ml)	6.75 (13.5/b)	hydrolyzed whey protein, hydrolyzed lactalbumin, hydrolyze	1.5/b (2.2 : 1.1 : 1)	0.4/b 1.12/b	100%	-	-	5.5 (11/b)	0.12 (0.24/b) (R = 2.2)	3.85 (7.7/b) (70%)	18.4 (36.8/b)	0	-	liquid vanilla,berries,coffee	487 mOsmol/l	brick HDPE 2 plastic Top HDPE 2 plastic Label PETG 7 plastic Closing film C/ALU 90 aluminum External box PAP 21 paper

				d Na caseinate														
n.73 FSMP	220 ml	127 (279/b) (1.27 kcal/ml)	6.65 (14.6/b)	concentrated and/or isolated whey protein, Na caseinate	1.45/b (1.9 : 1.2 : 1)	0.57/b 1.63/b	100%	-	-	2.56 (5.63/b)	0.04 (0.09/b) EPA + DHA = 0.65 (1.43/b) (R = 0.3)	0.42 (0.93/b) (16.4%)	18.33 (40.3/b)	2.07 (4.55/b) of which 1.1 (/b) soluble; FOS	-	liquid chocolate,blackberry,banana-vanilla	597 mOsmol/l	brick HDPE 2 plastic Top HDPE 2 plastic Label PETG 7 plastic Closing film C/ALU 90 aluminum External box PAP 21 paper
n.74 FSMP	220 ml	93 (204.6/b) (0.93 kcal/ml)	4.3 (9.4/b)	Na caseinate, isolated soy protein	0.8/b (1.8 : 1.2 : 1)	0.44/b 0.92/b	70%	30%	2.3	3.5 (7.7/b)	0.23 (0.51/b) (R = 2.7)	-	10.87 (23.9/b)	2.25 (4.95/b) of which 0.45 (0.99/b) soluble; FOS	low (42 ± 8)	liquid strawberry,vanilla,chocolate	610 mOsmol/l	brick HDPE 2 plastic Top HDPE 2 plastic Label PETG 7 plastic Closing film C/ALU 90 aluminum External box PAP 21 paper
n.75 FSMP	220 ml	150 (330/b) (1.5 kcal/ml)	7.5 (16.5/b)	Na and Ca caseinate, isolated	1.45/b (1.9 : 1.2 : 1)	0.68/b 1.61/b	80%	20%	4	7.5 (16.5/b)	0.42 (0.92/b) (R = 2.8)	-	12.75 (28/b)	1.5 (3.3/b) of which 1 (2.2/b)	low (25 ±3)	liquid strawberry,vanilla,coffee	671 mOsmol/l	-

				soy protein										soluble; FOS					
n.76 FSMP	220 ml	161 (354.2/b) (1.61 kcal/ml)	8.3 (18.3/b)	Na and Ca caseinate, isolated soy protein	1.58/b (1.7 : 1.2 : 1) β-HMB = 0.77/b	1.32/b 1.69/b	81.6%	18.4%	4.4	8.28 (18.2/b)	0.47 (1/b) (R = 3)	-	12.75 (28/b)	1.9 (4.2/b) of which 1 (2.2/b) soluble; FOS	low (-)	liquid strawberry,vanilla,coffee	704 mOsmol/l	brick HDPE 2 plastic Top HDPE 2 plastic Label PETG 7 plastic Closing film C/ALU 90 aluminum External box PAP 21 paper
n.77 FSMP	5 g	364 (18.2/s)	92.5 (4.6/s)	isolated whey protein	- -	- -	100%	-	-	≤ 0.05	-	-	< 0.06	0	-	powder, neutral	-	-
n.78 FSMP	5.85 g	402 (22.9/s)	0 4.5 g aa/s	EAA	1.2/s (2:1:1)	- -	-	-	-	0	-	-	36.8 (2.1/s)	0	-	powder, neutral	-	-
n.79 FSMP	-	-	0 11.5 g aa/s	EAA	1.2/s (2:1:1)	- 7/s	-	-	-	-	-	-	-	0	-	powder, neutral	-	-
n.80 supplement	5.5 g	374.5 (20.6/s)	0 4 g aa/s	EAA	1.25/s (2:1:1)	- -	-	-	-	0.15/s	-	-	0.25/s	0	-	powder, neutral	-	-
n.81 FSMP	5.5 g	382 (21/s)	0 4 g aa/s	EAA	1.25/s (2:1:1)	- -	-	-	-	1.09 (0.06/s)	-	-	6.54 (0.36/s)	0	-	powder, neutral	340 mOsmol/l (1 sach	-

																	et in 100 ml of water)	
n.82 FSMP	7 g	206 (24.8/s)	0<br>4 g aa/s	EAA	1.25/s (2:1:1)	-<br>-	-	-	-	0	-	-	5.6/s	0.9/s; -	-	gelling powder, neutral	-	-
n.83 FSMP	6.5 g	360 (23.4/s)	0<br>4 g aa/s	EAA	1.25/s (2:1:1) β-HMB = 0.75 g/s	-<br>-	-	-	-	0.06/s	-	-	1.8/s	0.1/s	-	powder, neutral	-	-
n.84 FSMP	25 g	408 (102/s)	0<br>12.5 g aa/s	EAA	1.56/s (2:1:1)	-<br>7/s	-	-	-	0.18/s	-	-	11.6/s	0	-	powder, neutral	-	-
n.85 FSMP	25 g	276 (69/s)	0<br>12.5 g aa/s	EAA	1.56/s (2:1:1)	-<br>7/s	-	-	-	0	-	-	10.2/s	0	-	gelling powder, neutral	-	-
n.86 FSMP	5.5 g	253 (13/s)	0<br>2.5 g aa/s	EAA	1.25/s (2:1:1) β-HMB = 0.75/s	-<br>-	-	-	-	0	-	-	1.3/s	0	-	powder, neutral	-	-

n.87 FSMP	5.5 g	193 (11/s)	0<br>2.5 g aa/s	EAA	1.25/s (2:1:1)<br>β-HMB = 0.75/s	-<br>-	-	-	-	0	-	-	2.1/s	1	-	powder, neutral	-	-
n.88 supplement	5 g	351 (17.6/s)	0<br>5 g aa/s	EAA	-<br>-	-<br>5/s	-	-	-	0	-	-	0	0	-	powder, neutral	-	-
n.89 supplement	5 g	275	0<br>5 g aa/p	BCAA	2.3/p (1.5 : 0.8 : 1)	-<br>-	-	-	-	0	-	-	0	0	-	powder, neutral	-	-
n.90 supplement	5 g	384 (19.2/p)	0	-	-	-	-	-	-	0	-	-	96 (4.8/p)	0	-	powder, neutral	-	-
n.91 supplement	2.5 g	368 (9.2/p)	87.2 (2.2/p)	concentrated milk protein	-<br>-	-<br>-	-	-	-	1.6 (0.04/p)	-	-	< 1.5 (0.03/p)	0	-	powder, neutral	25 mOsmol/l	-
n.92 supplement	5.7 g	20/s	0<br>5 g aa/s	BCAA	2/p (1.3 : 1 : 1)	-<br>-	-	-	-	0	-	-	0	0	-	powder, lemon,elderberry flavored,with stevia	-	-
n.93 FSMP	30 g	349 (105/p); 352	33.99 (10.2/p); 34.9	concentrated	-<br>-	-<br>-	-	-	-	2.54 (0.76/p);	-	-	45.5 (13.65/p);	4.37 (1.31/p); 4.61	-	powder, cocoa,vanilla, neutral	-	-

		(106/p) for chocolate	(10.47/p) for chocolate	whey protein						3.4 (1.02/p) for chocolate			43.2 (12.95/p)	(1.38/p) for chocolate; inulin				
n.94 FSMP	10 g	326 (33/s)	80 (8/s)	isolated whey protein	-<br>-	-<br>-	100%	-	-	0	-	-	1.5 (0.15/s)	0	-	powder, neutral	-	-
n.95 FSMP	10 g	364 (36.4/s)	92 (9.2/s)	isolated whey protein	0.99/s (1.5 : 1.1 : 1)	0.19/s<br>-	100%	-	-	< 1 (< 0.1/s)	-	-	< 1 (< 0.1/s)	0	-	powder, neutral, chocolate	-	-
n.96 FSMP	20 g	506 (101/p)	0.6 (0.1/p)	whey protein isolate	-<br>-	-<br>-	100%	-	-	30 (6/p)	1.7 (0.3/p) (R = 5.33)	-	59 (12/p)	0	-	powder, neutral	-	-
n.97 FSMP	10 g	384 (38/p)	0	-	-	-	-	-	-	0	-	-	96 (9.6/p)	0	-	powder, neutral	85 mOsmol/l	-
n.98 FSMP	21 g	359 (75/s)	68.5 (14.4/s)	isolated whey protein BCAA	3.8/s (2.9 : 1.1 : 1) β-HMB = 1.34 g/s	-<br>-	100%	-	-	0.5 (0.11/s)	-	-	18.9 (4/s)	2.3 (0.48/s); -	-	powder, cookie	-	-

n.99 FSMP	5.5 g	302 (16.6/s)	73.48 (4.04/s) 2.5 g BCAA	aa BCAA	1.25/s (2 : 1 : 1)	0.5/s -	-	-	-	0	-	-	2 (0.11/s)	0	-	powder, red fruits	-	-
n.100 FSMP	5.5 g	328.2 (18/s)	74.2 (4.1/s) 2.5 g BCAA	aa BCAA	1.25/s (2 : 1 : 1)	0.5/s 0.1/s	-	-	-	0	-	-	3.64 (0.2/s)	0	-	powder, orange	-	-
n.101 FSMP	10 g	314 (31.4/s)	42.1 (4.21/s) 2.5 g BCAA	aa BCAA	1.25/s (2 : 1 : 1)	0.5/s 0.1/s	-	-	-	0	-	-	27.6 (2.76/s)	13.1 (1.31/s)	-	powder orange	-	-
n.102 FSMP	5 g	350 (18/s)	87.5 (4.4/s)	aa	- -	- 5/s	-	-	-	0	-	-	0	-	-	powder, neutral	-	-
n.103 FSMP	15 g	362 (54/p)	88 (13/p)	whey protein	0.47/s (1.6 : 0.9 : 1)	0.07/s -	100%	-	-	1 (0.2/p)	-	-	0.3 (0.0/s)	0	-	powder, neutral	-	-jar paper/cardboard C/PAP 85 . recycle -cover plastic P 5 . recycle - film inside aluminium ALU 41 . recycle
n.104 FSMP	15 g	371 (57/s)	90 (13.5/s)	whey protein	1.5/s (1.7 : 1 : 1)	0.6/s -	100%	-	-	1 (0.15/s)	-	-	0.5 (0.075/s)	0	-	powder, neutral	-	-jar paper/cardboard C/PAP 85 . recycle -cover plastic P 5 . recycle - film inside aluminium ALU 41 . recycle

																		- external box paper PAP 21 recycle
n.105 FSMP	34 g	385 (131/p)	22.6 (7.7/p)	milk protein	-<br>-	-<br>-	100%	-	-	3.8 (1.3/p)	-	-	65.1 (22.1/p)	0	-	powder, neutral	-	Jarpaper/cardboard C/PAP 85 . recycle<br>Top Plastic PP 5 . recycle<br>film inside aluminium ALU 41 . recycle
n.106 supplement	12 g	360 (43.2/p)	90 (10.8/p)	collagen peptides	-<br>-	-<br>-	100%	-	-	0	-	-	0	0	-	powder, neutral	-	-
n.107 FSMP	12 g	365 (43.8/p)	87.9 (10.5/p)	whey protein	1.2/p (1.6 : 0.89 : 1)	0.25/s<br>-	100%	-	-	1.3 (0.156/p)	-	-	0.4 (0.048/p)	0	-	powder, neutral	-	-
n.108 supplement	5 g	369 (18.4/p)	88.6 (4.4/p)	casein	0.5/p (1.8 : 1.3 : 1)	0.19/s<br>1.18/s	100%	-	-	1 (0.05/p)	-	-	0	0	-	powder, neutral	-	-
n.109 supplement	7 g	380 (26.6/p)	0	-	-	-	-	-	-	0	-	-	95 (6.65/p)	0	-	powder, neutral	-	-
n.110 supplement	5 g	400 (20/s)	0<br>5 g aa/s	aa	-<br>-	-<br>5/s	-	-	-	0	-	-	0	-	-	powder, neutral	-	-
n.111 FSMP	24 g for ora	372 (89/s) for orange	0<br>14.8 g di aa/s	aa	-<br>-<br>β-HMB	7.4/s<br>7.4/s	-	-	-	0.1 (0.02/s)	-	-	27.1 (6.5/s) for orange	0	-	powder, neutral, orange	386-407 mOs	-

	nge ; 19.3 g for neutral flavour	; 384 (74/s) for neutral			= 1.3 g/s								; 14 (2.7/s) for neutral				mol/l	
n.112 FSMP	76 g	406.6 (309/s)	20.8 (15.8/s)	whey protein, isolated soy protein	1.2/s (1.8 : 1.2 : 1)	1.3/s<br>4.3/s	75.2%	24.8%	3	4,5/s	-	2.4/s (53%)	51.6/s	0	-	powder, vanilla	504 mOsmol/l	-
n.113 supplement	53.5 g	233/p	8.65/p	Ca caseinate, isolated soy protein	0.8/p (1.78 : 1.2 : 1) β-HMB = 1.5 g/p	0.47/p<br>-	70%	30%	2.3	7.6/p	0.27 g/p	-	30.6/p	1.6/p, soluble; FOS	-	powder, vanilla,strawberry,chocolate	460 mOsmol/l	-
n.114 FSMP	6.3 g	52 (3/s)	59.7 (3.76/s) 4.3 g aa/s	EAA	1,01 g (1.3 : 0.9 : 1)	-<br>-	-	-	-	0	-	-	4.3 (< 0.5/s)	0	-	powder, citrus fruits	-	-
n.115 FSMP	6.5 g	61 (4/s)	64.7 (4.2/s) 5 g aa/s	BCAA	2.5/s (2 : 1 : 1)	-<br>-	-	-	-	0	-	-	9.5 (0.6/s)	0	-	powder, citrus fruits	291 mOsmol/l	-

n.116 FSMP	6 g	356 (21/p)	86 (5/p)	Ca caseinate	-<br>-	-<br>-	100%	-	-	1 (0/p)	-	-	0.3 (0/p)	0	-	powder, neutral	24 mOsmol/l	-
n.117 FSMP	10 g	355 (36/s)	86 (8.6/s)	whey protein	-<br>-	-<br>-	100%	-	-	< 1 (< 0.1/s)	-	-	1 (0.1/s)	0	-	powder, neutral	33 mOsmol/l	-
n.118 FSMP	12.5 g	360 (90/p)	35 (9/p)	whey protein, isolated pea protein	-<br>-	-<br>-	-	-	-	0.25 (0.1/s)	-	-	54 (13/s)	0	-	powder, neutral,vanilla,chocolate	-	-
n.119 FSMP	25 g	140 (35/s)	64.3 (16.1/s) 4 g aa/s	EAA Arg Glutamine	1.25 (2 : 1 : 1) β-HMB = 1.5/s	4/s<br>4/s	-	-	-	0	-	-	25 (6/s)	0	-	powder, citrus fruits	447 mOsmol/l	-
n.120 FSMP	50 g	404 (200/s)	22 (11/s)	Glutamine	-<br>-	-<br>3.5/s	-	-	-	6.1 (3/s)	-	-	66 (33/s)	0	-	powder, vanilla	480 mOsmol/l	-
n.121 FSMP	5 g	351 (18/s)	22 (11/s)	Glutamine	-<br>-	-<br>5/s	-	-	-	0	-	-	0	0	-	powder, neutral	182 mOsmol/l	-
n.122 FSMP	15 g	498 (149/p)	0	-	-	-	-	-	-	23 (7/p)	-	90-100%	73 (22/p)	0	-	powder, neutral	128 mOsmol/l	-

n.123 FSMP	15 g	380 (57/p)	0	-	-	-	-	-	-	0	-	-	95 (14/p)	0	-	powder, neutral	80 mOsmol/l	-
n.124 FSMP	220 ml	180 (396/b) (1.8 kcal/ml)	4.52 (9.94/b)	isolated milk protein, Na-caseinate	0.88/b (1.8 : 1.2 : 1)	0.33/b 1.06/b	100%	-	-	9.7 (21.3/b)	0.27 (0.59/b) (R = 4.8)	-	18.5 (40.8/b)	2.78/b, mainly soluble (1.94 g FOS/b)	44	liquid, vanilla	590 mOsmol/l	(plastic brick)
n.125 FSMP	220 ml	180 (396/b) (1.8 kcal/ml)	8.1 (17.8/b)	caseinates, isolated milk protein	1.58/b (1.8 : 1.2 : 1)	0.61/b 1.8/b	100%	-	-	9.77 (21.5/b)	0.27 (0.59/b) (R = 4.8)	-	14.7 (32.4/b)	2.77/b, mainly soluble (1.84 g FOS/b)	35	liquid, strawberry,vanilla	538 mOsmol/l	(plastic brick)
n.126 FSMP	250 ml	150 (375/b) (1.5 kcal/ml)	6.25 (15.6/b)	caseinates	1.4/b (2 : 1.3 : 1)	0.5/b 1.5/b	100%	-	-	9.33 (23.3/b)	0.49 (1.22/b) (R = 3.9)	1.87 (4.66/b) (20%)	10.6 (26.4/b)	0	-	liquid, vanilla	383 mOsmol/l	-
n.127 FSMP	125 ml	200 (250/b) (2 kcal/ml)	4 (5/b)	whey protein	- -	- -	100%	-	-	10 (12.5/b)	0.2 (0.25/b) (R = 8)	-	23.5 (29.4/b)	0	-	liquid, apricot	455 mOsmol/l	(plastic)
n.128 FSMP	125 ml	200 (250/b) (2 kcal/b)	7.3 (9.1/b)	whey protein	- -	- -	100%	-	-	10 (12.5/b)	0.2 (0.25/b) (R = 8)	-	20 (25/b)	0	-	liquid, caramel,apricot	410 mOsmol/l	(plastic)

n.129 FSMP	125 ml	150 (188/b) (1.5 kcal/ml)	7.5 (9.4/b)	milk protein	- -	- -	100%	-	-	3.3 (4.1/b)	0.1 (0.12/b) (R = 5)	-	22.6 (28.2/b)	0	-	liquid, vanilla,strawberry,chocolate	425-435-465 mOsmol/l	(plastic)
n.130 FSMP	200 ml	200 (400/b) (2 kcal/ml)	7 (14/b)	casein, whey protein	- -	- -	100%	-	-	9.6 (19.2/b)	0.38 (0.76/b) (R = 3.74)	-	21 (42/b)	3.2/b, mainly soluble (3 g/b FOS)	-	liquid, vanilla	430 mOsmol/l	(plastic)
n.131 FSMP	500 ml	130 (650/b) (1.3 kcal/ml)	6.5 (32.5/b)	casein, soy protein	- -	- -	-	-	-	5.8 (29/b)	0.21 (1.05/b) (R = 6.6)	3 (15/b) (52%)	13 (65/b)	< 0.1 (< 0.5/b)	-	liquid, neutral	335 mOmsol/l	(plastic)
n.132 FSMP	200 ml	200 (400/b) (2 kcal/ml)	3 (6/b)	milk protein	- -	- -	100%	-	-	8.9 (17.8/b)	- EPA+DHA = 0.06 (0.12/b)	2.2 (4.4/b) (25%)	26.4 (52.8/b)	1.2 (2.4/b), soluble (tapioca dextrins)	-	liquid, vanilla,cappuccino	500-565 mOsmol/l	(plastic)

Supplementary Table 2. Classification of the analyzed FSMPs and supplements according to their micronutrients content. Abbreviations used: n.: number; Ca: Calcium; Mg: Magnesium; Fe: Iron; Zn: Zinc; Se: Selenium; Cr: Chromium; vit: vitamin; FSMP: food for special medical purpose. Information was gathered from the companies' compedia and from their official websites.

**Product n. and type**	**Micronutrients**													
	**Minerals**						**Vitamins**							
	**Ca (mg/p)**	**Mg (mg/p)**	**Fe (mg/p)**	**Zn (mg/p)**	**Se (μg/p)**	**Cr (μg/p)**	**vit A (μg-RE/p) carotenoids (mg/p)**	**vit D (μg/p)**	**vit E (mg-α-TE/p)**	**vit B1 (mg/p)**	**vit B6 (mg/p)**	**folic acid (μg/p)**	**vit B12 (μg/p)**	**vit C (mg/p)**
n.1 FSMP	500	37.2-37.4	2.49-2.51	2.24	15.7	10.9	151-152<br>-	20	7.5-7.57	0.23	0.75	202-203	3.06-3.11	32.4
n.2 FSMP	560	84-86	6	4.6	22	26-36	376<br>-	4.6	4.6	0.56	0.66	100	1.4	38
n.3 FSMP	400	60.6	2.5	2.24	18.34	8.86	150-152<br>-	10	4.8	0.26	0.76	80	1.16	32
n.4 FSMP	182	46	4.8	3.6	17.2	30	246<br>carotenoids 0.6	2.2	3.8	0.46	0.52	80	0.64	30
n.5 FSMP	106	46	3.2	2.4	15	24	164<br>carotenoids 0.4	2.4	5	0.8	0.6	76	1.3	30
n.6 FSMP	217.5-220	40.6-41.2	4.8-4.81	3.6	17.5-18.3	20	300<br>-	2.25	3.75	0.5	0.5	80	0.88	30
n.7 FSMP	217.5	41.2	4.8	3.6	17.5	20	300<br>-	2.25	3.75	0.5	0.5	80	0.62	30
n.8 FSMP	225	50	3.75	3.6	17.5	20	300<br>-	2.12	3.75	0.45	0.52	80	0.62	30
n.9 FSMP	88.5	19.5	1.3	1.3	9	9	97.5<br>-	1.35	1.3	0.15	0.2	25.5	0.66	21
n.10 FSMP	437.5	67.5	2.7	3.2	19.2	16.2	325<br>-	2.6	6.1	0.65	0.76	101.1	1.1	38.4
n.11 FSMP	250	35	4.14	2.86	17.5	8.76	187.6<br>-	5.38	4.9	0.3	0.42	82.6	1	27.6

n.12 FSMP	446	180	6	7	46	22	420<br>-	6	12	0.8	1.1	210	1.6	60
n.13 FSMP	450	84	6	9	64	26	238 carotenoids 1.5	2.6	38	0.56	1.3	200	1.58	250
n.14 FSMP	410	56 (36 for chocolate flavour)	5	4	27	25	426 β-carotene 0.126	5	7.5	0.6	0.86	125	1.5	37.6
n.15 FSMP	410	32	5	3.2	27	25	425 β-carotene 0.126	10	7.5	0.6	0.86	125	1.5	37.6
n.16 FSMP	245	20	3.1	2.5	17.5	16	266 β-carotene 0.078	6	2.5-4.69	0.4	0.54	78.1	0.94	23.5
n.17 FSMP	206.2	50	6.5	4.6	28.8	28.8	402.5 β-carotene 0.115	10	8.6	0.59	0.75	118.8	1.38	52.5
n.18 FSMP	-	-	-	-	-	-	-<br>-	-	16.8	-	-	-	-	-
n.19 FSMP	300	30	4	3	20	20	340 β-carotene 0.100	4	6	0.46	0.66	100	1.2	30
n.20 FSMP	700	32	4	4.8	28	25	520<br>-	10	8	0.86	0.94	160	2.2	60
n.21 FSMP	437.5	20	2.5	3	17.5	15.6	325<br>-	6.2	5	0.86	0.59	100	1.38	37.5
n.22 FSMP	180	54	3.4	2.4	16.6	32	294<br>-	3.6	4	0.36	0.44	90	1.1	30
n.23 FSMP	220-224	60-64	3.6	3.6	22	22	270 β-carotene 0.3	3	6	0.6	0.6	90	0.9	30
n.24 FSMP	150	40	2.4	2.4	14	20	180	2	3	0.4	0.4	60	0.6	20

							β-carotene 0.2							
n.25 FSMP	250	70	4.6	4.0	20	20	320 β-carotene 0.4	5	7	0.6	0.8	160	1.2	40
n.26 FSMP	280	66	4.8	4.0	16.6	26	334 β-carotene 0.3	5.8	5	0.4	0.6	110	1.1	36
n.27 FSMP	56	17.2	0.4	0.2	10	3	40 -	10	4.2	-	-	24	1	-
n.28 FSMP	440	46	2	4	18	20	280 -	2.8	5.6	0.46	0.64	90	1.2	26
n.29 FSMP	440	50	3	4	20	26	280 -	3.2	8	0.46	0.64	90	1.2	36
n.30 FSMP	460	42	3	4.4	20	26	260 -	3.2	8	0.36	0.64	90	1.2	30
n.31 FSMP	420	36	3.2	4	20	26	260 -	3.2	7.4	0.36	0.64	90	1.2	30
n.32 FSMP	37.6	35	3.4	3	20	22	- -	4.2	4.2	0.64	0.62	70.4	1	-
n.33 FSMP	288	12.5 (31.5 for chocolate)	0.11 (1.4 for chocolate)	1.5	7.3-7.5	12.5	175 -	1.8	4	0.29	0.36-0.4	56.3 (50 for chocolate)	0.3-0.5	25
n.34 FSMP	238	18.8	-	-	-	-	175 -	1.8	2.5	0.29	0.4	56.3	0.44	16.3
n.35 FSMP	281	30 (25 for vanilla)	3.1 (2 for vanilla)	2.8	5.3	19	160 -	2	4.5	0.43	0.5	62.5	0.25	22.5
n.36 FSMP	270	76	4	5	15.6	33	329 -	2.2	10	0.4	0.5	66	1.9	71
n.37 FSMP	100	40	2.2	2	13	13	198 -	2.2	3	0.3	0.42	56	0.5	28
n.38 FSMP	231	31	1.8	3	15	19	225 -	2	4.7	0.44	0.62	50	0.62	37

n.39 FSMP	480	56	3.6	4	40	20	430<br>-	14	7.2	0.52	1.1	140	2.2	32
n.40 FSMP	-	-	-	-	-	-	-<br>-	-	-	-	-	-	-	-
n.41 FSMP	300	31	3.9	3.2	20	20	250<br>-	3.1	6.2	0.85	0.7	90	0.95	37
n.42 FSMP	160	56	3.4	3.4	16	15	276<br>-	3.6	6	0.46	0.7	90	0.44	30
n.43 FSMP	250	50	3.4	4	19	16	240<br>-	2.8	5.4	0.3	0.48	78	1.14	40
n.44 FSMP	380	40	3	3.4	24	13	330<br>-	3.6	8	0.4	0.7	80	0.8	34
n.45 FSMP	264	44	2.4	2.8	20	14	192<br>-	2.6	5.2	0.32	0.4	58	1.2	44
n.46 FSMP	220	38	3.6	8	24	12	224<br>β-carotene<br>0.230	3	12	0.5	0.58	60	0.88	230
n.47 FSMP	240	-	3	2.6	15	11	240<br>-	2.6	4.6	0.4	0.5	70	1.3	32
n.48 supplement	330	66	4.5	4.8	17.1	-	330<br>-	9.9	3.6	0.36	0.72	120	1.35	25
n.49 fortified food	150	-	-	2.5	13	19	269<br>-	1.8	4.8	0.38	0.6	45	-	30
n.50 FSMP	-	-	-	-	-	-	320<br>-	4	4.6	0.5	0.6	90	1	34
n.51 fortified food	-	-	-	-	-	-	-<br>-	-	-	-	-	-	-	-
n.52 FSMP	270	36	2.4	2.2	10	14	130<br>-	2	3	0.24	0.4	40	0.7	16

n.53 FSMP	206	21	1.1	1.1	8.7	6.2	237 -	1.2	3.7	0.31	0.44	40	0.37	9
n.54 FSMP	336	60 (70 for chocolat e)	4.6	4	28.6	22.6	218 -	6.2	11.6	0.8	1	120.4	1.6	56
n.55 FSMP	336	60	4.6	4	28	22	218 -	6.2	7.6	0.8	1	120.4	1.5	56
n.56 FSMP	200	1.04	39.1	3.11	22	17.3	154 -	4.3	5.4	0.54	0.77	85	1.08	40
n.57 FSMP	120	52	3.8	3.4	24	18.8	171 -	4.8	6	0.6	0.86	94	1.2	44
n.58 FSMP	320	58	4.2	3.8	27	21	256 -	7.2	8.8	0.9	1.3	141	1.8	65
n.59 FSMP	160	40	3	2.8	19	15	170.8 -	4.8	6	0.6	0.86	94	1.2	44
n.60 FSMP	160	50	3.8	3.4	24	18.8	214 -	6	7.4	0.76	1.06	117.6	1.5	54
n.61 FSMP	230	60	4.6	4.4	28	22	400 -	7.2	8.8	0.9	1.28	141	2	66
n.62 FSMP	183-205-218	-	-	-	-	-	- -	-	-	-	-	-	-	-
n.63 FSMP	286	-	-	-	-	-	- -	-	-	-	-	-	-	-
n.64 fortifi ed food	-	-	-	-	-	-	- -	-	1.6-1.8-2	-	-	-	-	-
n.65 FSMP	191	-	-	-	-	-	- -	-	-	-	-	-	-	-
n.66 fortifi ed food	22.4-29.4	1.9-20.4	-	6.3-6.4	-	-	163-169-175 -	-	43-44	-	-	-	-	86-116

n.67 FSMP	234	60	4.2	3.2	16.6	15	176<br>β-carotene<br>0.058	3.4	4.8	0.4	0.54	66	1.3	24
n.68 FSMP	125	34.1	2.38	1.8	9.5	8.5	100<br>β-carotene<br>0.032	1.91	2.75	0.22	0.31	37.5	0.74	13.6
n.69 FSMP	499.4	55	4.62	3.85	19.8	18.7	264<br>β-carotene<br>0.132	12.5	5.5	0.57	0.66	77	1.43	35.2
n.70 FSMP	300	80	4.2	4.8	18	20	316<br>-	3.4	6.8	0.6	0.68	96	1.28	40
n.71 FSMP	212.5	25	4.25	3.6	15	12.5	193.8<br>-	3.75	5.88	0.44	0.59	60	1.21	26.2
n.72 FSMP	200	60	4	3.6	19	16	300<br>-	2	3.8	0.42	0.6	60	1	36
n.73 FSMP	220	92.4	1.43	5.5	17.4	19.8	297<br>β-carotene<br>0.154	3.74	44	0.55	0.748	70.4	0.77	94.6
n.74 FSMP	187	39.6	2.86	2.42	13.2	11	220<br>-	1.39	3.96	0.37	0.57	55	0.88	19.8
n.75 FSMP	220	68.2	1.87	3.74	20.9	19.8	316.8<br>-	5.5	6.64	0.57	0.86	88	1.1	28.6
n.76 FSMP	270.6	57.2	2.64	3.74	20.9	18.5	316.8<br>-	7	6.6	0.57	0.86	88	1.1	28.6
n.77 FSMP	25	-	-	-	-	-	-<br>-	-	-	-	-	-	-	-
n.78 FSMP	-	-	-	-	-	-	-<br>-	-	-	0.7	0.85	-	-	-
n.79 FSMP	-	-	-	-	-	-	-<br>-	-	-	-	-	-	-	-
n.80 suppl ement	-	-	-	-	-	-	-<br>-	-	-	0.15	0.15	-	-	-

n.81 FSMP	-	-	-	-	-	-	-<br>-	-	-	-	-	-	-	-
n.82 FSMP	-	-	-	-	-	-	-<br>-	-	-	0.15	0.15	-	-	-
n.83 FSMP	-	-	-	-	-	-	-<br>-	10	-	0.15	0.15	-	-	-
n.84 FSMP	-	-	-	-	-	-	-<br>-	-	-	0.15	0.15	-	-	15
n.85 FSMP	-	-	-	-	-	-	-<br>-	-	-	0.15	0.15	-	-	15
n.86 FSMP	-	-	-	5	-	-	-<br>-	-	-	-	-	-	-	-
n.87 FSMP	-	-	-	5	-	-	-<br>-	20	-	-	-	-	-	-
n.88 supplement	-	-	-	-	-	-	-<br>-	-	-	-	-	-	-	-
n.89 supplement	-	-	-	-	-	-	-<br>-	-	-	-	-	-	-	-
n.90 supplement	-	-	-	-	-	-	-<br>-	-	-	-	-	-	-	-
n.91 supplement	33.8	≤ 0.5	-	-	-	-	-<br>-	-	-	-	-	-	-	-
n.92 supplement	-	-	-	-	-	-	-<br>-	-	-	-	-	-	-	-
n.93 FSMP	198	66	4.8	1.5	7.2	-	180<br>-	2.4	3.6	0.36	0.72	87	0.75	21
n.94 FSMP	66	22	1.6	0.5	2.4	-	60<br>-	0.8	1.2	0.12	0.24	29	0.25	7
n.95 FSMP	-	-	–	-	-	-	-<br>-	-	-	-	-	-	-	-

n.96 FSMP	-	-	-	-	-	-	- -	-	-	-	-	-	-	-
n.97 FSMP	0.1	-	-	-	-	-	- -	-	-	-	-	-	-	-
n.98 FSMP	40.9	4.9	-	-	-	-	- -	10	-	-	1.7	-	-	-
n.99 FSMP	-	-	-	-	-	-	- -	-	-	0.15	0.15	-	-	-
n.100 FSMP	-	-	-	7.5	-	-	- -	10	-	0.25	0.2	-	-	35
n.101 FSMP	-	-	-	7.5	-	-	- -	10	-	0.25	0.2	-	-	35
n.102 FSMP	-	-	-	-	-	-	- -	-	-	-	-	-	-	-
n.103 FSMP	30	-	-	-	-	-	- -	-	-	-	-	-	-	-
n.104 FSMP	70	-	-	-	-	-	- -	-	-	-	-	-	-	-
n.105 FSMP	170	24	1.2	1.3	8.2	4.1	136 -	1.4	1.4	0.31	0.27	25.5	0.5	15.3
n.106 supplement	-	-	-	-	-	-	- -	-	-	-	-	-	-	-
n.107 FSMP	69.96	1.5	-	-	-	-	- -	-	-	-	-	-	-	-
n.108 supplement	-	-	-	-	-	-	- -	-	-	-	-	-	-	-
n.109 supplement	-	-	-	-	-	-	- -	-	-	-	-	-	-	-
n.110 supplement	-	-	-	-	-	-	- -	-	-	-	-	-	-	-

n.111<br>FSMP	215	-	-	-	-	-	-<br>-	-	-	-	-	-	-	-
n.112<br>FSMP	21.9	5.04	0.39	0.28	1.14	1.38	32.1<br>-	0.52	0.2	0.04	0.07	8.11	0.09	3.66
n.113<br>suppl<br>ement	321	31.4	1.31	1.4	12	12.5	256<br>β-carotene<br>0.011	5	4.3	0.43	0.55	71	0.81	29.4
n.114<br>FSMP	-	-	-	-	-	-	-<br>-	-	-	0.15	0.15	-	-	-
n.115<br>FSMP	-	-	-	-	-	-	-<br>-	-	-	0.3	0.3	-	-	-
n.116<br>FSMP	-	-	-	-	-	-	-<br>-	-	-	-	-	-	-	-
n.117<br>FSMP	-	-	-	-	-	-	-<br>-	-	-	-	-	-	-	-
n.118<br>FSMP	75	40	4	2	-	-	225<br>-	2	3	0.2	0.5	-	0.7	11
n.119<br>FSMP	-	-	-	-	-	-	-<br>-	-	-	0.22	0.28	-	-	-
n.120<br>FSMP	129	15	1	1	5	25.2	107.5<br>-	3	3.5	0.56	0.58	60	0.77	22.4
n.121<br>FSMP	-	-	-	-	-	-	-<br>-	-	-	-	-	-	-	-
n.122<br>FSMP	-	-	-	-	-	-	-<br>-	-	-	-	-	-	-	-
n.123<br>FSMP	-	-	-	-	-	-	-<br>-	-	-	0.07	0.1	-	-	-
n.124<br>FSMP	161	46.2	4.18	4.18	16.7	27.9	209<br>-	2.64	7.48	0.92	1.1	132	1.87	24.2
n.125<br>FSMP	233	46.2	4.18	4.18	16.3	9.9	209<br>-	2.64	7.48	0.92	1.1	132	1.87	23.1
n.126<br>FSMP	250	75	5	4.2	19.2	25	207.5<br>β-carotene<br>0.168	2.75	7.5	0.62	0.88	95	1.25	37.5

n.127 FSMP	7.5	< 0.75	4	3.4	20	16.2	0 carotenoids 0.56	1.75	6.25	0.375	0.75	125	0.59	7.5
n.128 FSMP	11.2	< 1.25	2.5	2.5	20	15	0 carotenoids 0.56	0	6.25	0.375	1.25	125	0.59	7.5
n.129 FSMP	193.8	18.8	4	3	28.8	16.2	158.8 carotenoids 0.75	1.75	6.25	0.375	0.44	66.2	0.625	50
n.130 FSMP	274	42	3.8	4	20	20	320 β-carotene 0.4	5	7	0.6	0.8	160	1.2	40
n.131 FSMP	490	130	8	8	45.5	45.5	585 β-carotene 0.65	6.5	10	1.3	1.3	195	1.95	65
n.132 FSMP	168	40	4	3.6	20	6	162.6 β-carotene 0.066	2	6	0.52	1.34	200	1.2	20

Supplementary Table S2. Classification of the analyzed FSMPs and supplements according to their micronutrients content. Abbreviations used: n.: number; Ca: Calcium; Mg: Magnesium; Fe: Iron; Zn: Zinc; Se: Selenium; Cr: Chromium; vit: vitamin; FSMP: food for special medical purpose. Information was gathered from the companies' compedia and from their official websites.

**Product n. and type**	**Micronutrients**													
	**Minerals**						**Vitamins**							
	**Ca (mg/p)**	**Mg (mg/p)**	**Fe (mg/p)**	**Zn (mg/p)**	**Se (μg/p)**	**Cr (μg/p)**	**vit A (μg-RE/p) carotenoids (mg/p)**	**vit D (μg/p)**	**vit E (mg-α-TE/p)**	**vit B1 (mg/p)**	**vit B6 (mg/p)**	**folic acid (μg/p)**	**vit B12 (μg/p)**	**vit C (mg/p)**
n.1 FSMP	500	37.2-37.4	2.49-2.51	2.24	15.7	10.9	151-152 -	20	7.5-7.57	0.23	0.75	202-203	3.06-3.11	32.4
n.2 FSMP	560	84-86	6	4.6	22	26-36	376 -	4.6	4.6	0.56	0.66	100	1.4	38
n.3 FSMP	400	60.6	2.5	2.24	18.34	8.86	150-152 -	10	4.8	0.26	0.76	80	1.16	32
n.4 FSMP	182	46	4.8	3.6	17.2	30	246 carotenoids 0.6	2.2	3.8	0.46	0.52	80	0.64	30
n.5 FSMP	106	46	3.2	2.4	15	24	164 carotenoids 0.4	2.4	5	0.8	0.6	76	1.3	30
n.6 FSMP	217.5-220	40.6-41.2	4.8-4.81	3.6	17.5-18.3	20	300 -	2.25	3.75	0.5	0.5	80	0.88	30

n.7 FSMP	217.5	41.2	4.8	3.6	17.5	20	300 -	2.25	3.75	0.5	0.5	80	0.62	30
n.8 FSMP	225	50	3.75	3.6	17.5	20	300 -	2.12	3.75	0.45	0.52	80	0.62	30
n.9 FSMP	88.5	19.5	1.3	1.3	9	9	97.5 -	1.35	1.3	0.15	0.2	25.5	0.66	21
n.10 FSMP	437.5	67.5	2.7	3.2	19.2	16.2	325 -	2.6	6.1	0.65	0.76	101.1	1.1	38.4
n.11 FSMP	250	35	4.14	2.86	17.5	8.76	187.6 -	5.38	4.9	0.3	0.42	82.6	1	27.6
n.12 FSMP	446	180	6	7	46	22	420 -	6	12	0.8	1.1	210	1.6	60
n.13 FSMP	450	84	6	9	64	26	238 carotenoids 1.5	2.6	38	0.56	1.3	200	1.58	250
n.14 FSMP	410	56 (36 for chocolate flavour)	5	4	27	25	426 β-carotene 0.126	5	7.5	0.6	0.86	125	1.5	37.6
n.15 FSMP	410	32	5	3.2	27	25	425 β-carotene 0.126	10	7.5	0.6	0.86	125	1.5	37.6
n.16 FSMP	245	20	3.1	2.5	17.5	16	266	6	2.5-4.69	0.4	0.54	78.1	0.94	23.5

							β-carotene 0.078							
n.17 FSMP	206.2	50	6.5	4.6	28.8	28.8	402.5 β-carotene 0.115	10	8.6	0.59	0.75	118.8	1.38	52.5
n.18 FSMP	-	-	-	-	-	-	- -	-	16.8	-	-	-	-	-
n.19 FSMP	300	30	4	3	20	20	340 β-carotene 0.100	4	6	0.46	0.66	100	1.2	30
n.20 FSMP	700	32	4	4.8	28	25	520 -	10	8	0.86	0.94	160	2.2	60
n.21 FSMP	437.5	20	2.5	3	17.5	15.6	325 -	6.2	5	0.86	0.59	100	1.38	37.5
n.22 FSMP	180	54	3.4	2.4	16.6	32	294 -	3.6	4	0.36	0.44	90	1.1	30
n.23 FSMP	220-224	60-64	3.6	3.6	22	22	270 β-carotene 0.3	3	6	0.6	0.6	90	0.9	30
n.24 FSMP	150	40	2.4	2.4	14	20	180 β-carotene 0.2	2	3	0.4	0.4	60	0.6	20
n.25 FSMP	250	70	4.6	4.0	20	20	320 β-carotene 0.4	5	7	0.6	0.8	160	1.2	40

n.26 FSMP	280	66	4.8	4.0	16.6	26	334<br>β-carotene 0.3	5.8	5	0.4	0.6	110	1.1	36
n.27 FSMP	56	17.2	0.4	0.2	10	3	40<br>-	10	4.2	-	-	24	1	-
n.28 FSMP	440	46	2	4	18	20	280<br>-	2.8	5.6	0.46	0.64	90	1.2	26
n.29 FSMP	440	50	3	4	20	26	280<br>-	3.2	8	0.46	0.64	90	1.2	36
n.30 FSMP	460	42	3	4.4	20	26	260<br>-	3.2	8	0.36	0.64	90	1.2	30
n.31 FSMP	420	36	3.2	4	20	26	260<br>-	3.2	7.4	0.36	0.64	90	1.2	30
n.32 FSMP	37.6	35	3.4	3	20	22	-<br>-	4.2	4.2	0.64	0.62	70.4	1	-
n.33 FSMP	288	12.5 (31.5 for chocolate)	0.11 (1.4 for chocolate)	1.5	7.3-7.5	12.5	175<br>-	1.8	4	0.29	0.36-0.4	56.3 (50 for chocolate)	0.3-0.5	25
n.34 FSMP	238	18.8	-	-	-	-	175<br>-	1.8	2.5	0.29	0.4	56.3	0.44	16.3
n.35 FSMP	281	30 (25 for vanilla)	3.1 (2 for vanilla)	2.8	5.3	19	160<br>-	2	4.5	0.43	0.5	62.5	0.25	22.5
n.36	270	76	4	5	15.6	33	329	2.2	10	0.4	0.5	66	1.9	71

FSMP							-							
n.37 FSMP	100	40	2.2	2	13	13	198<br>-	2.2	3	0.3	0.42	56	0.5	28
n.38 FSMP	231	31	1.8	3	15	19	225<br>-	2	4.7	0.44	0.62	50	0.62	37
n.39 FSMP	480	56	3.6	4	40	20	430<br>-	14	7.2	0.52	1.1	140	2.2	32
n.40 FSMP	-	-	-	-	-	-	-<br>-	-	-	-	-	-	-	-
n.41 FSMP	300	31	3.9	3.2	20	20	250<br>-	3.1	6.2	0.85	0.7	90	0.95	37
n.42 FSMP	160	56	3.4	3.4	16	15	276<br>-	3.6	6	0.46	0.7	90	0.44	30
n.43 FSMP	250	50	3.4	4	19	16	240<br>-	2.8	5.4	0.3	0.48	78	1.14	40
n.44 FSMP	380	40	3	3.4	24	13	330<br>-	3.6	8	0.4	0.7	80	0.8	34
n.45 FSMP	264	44	2.4	2.8	20	14	192<br>-	2.6	5.2	0.32	0.4	58	1.2	44
n.46 FSMP	220	38	3.6	8	24	12	224<br>β-carotene 0.230	3	12	0.5	0.58	60	0.88	230

n.47 FSMP	240	-	3	2.6	15	11	240<br>-	2.6	4.6	0.4	0.5	70	1.3	32
n.48<br>supplement	330	66	4.5	4.8	17.1	-	330<br>-	9.9	3.6	0.36	0.72	120	1.35	25
n.49 fortified food	150	-	-	2.5	13	19	269<br>-	1.8	4.8	0.38	0.6	45	-	30
n.50 FSMP	-	-	-	-	-	-	320<br>-	4	4.6	0.5	0.6	90	1	34
n.51 fortified food	-	-	-	-	-	-	-<br>-	-	-	-	-	-	-	-
n.52 FSMP	270	36	2.4	2.2	10	14	130<br>-	2	3	0.24	0.4	40	0.7	16
n.53 FSMP	206	21	1.1	1.1	8.7	6.2	237<br>-	1.2	3.7	0.31	0.44	40	0.37	9
n.54<br>FSMP	336	60 (70 for chocolate)	4.6	4	28.6	22.6	218<br>-	6.2	11.6	0.8	1	120.4	1.6	56
n.55<br>FSMP	336	60	4.6	4	28	22	218<br>-	6.2	7.6	0.8	1	120.4	1.5	56
n.56<br>FSMP	200	1.04	39.1	3.11	22	17.3	154<br>-	4.3	5.4	0.54	0.77	85	1.08	40
n.57	120	52	3.8	3.4	24	18.8	171	4.8	6	0.6	0.86	94	1.2	44

FSMP							-							
n.58 FSMP	320	58	4.2	3.8	27	21	256<br>-	7.2	8.8	0.9	1.3	141	1.8	65
n.59 FSMP	160	40	3	2.8	19	15	170.8<br>-	4.8	6	0.6	0.86	94	1.2	44
n.60 FSMP	160	50	3.8	3.4	24	18.8	214<br>-	6	7.4	0.76	1.06	117.6	1.5	54
n.61 FSMP	230	60	4.6	4.4	28	22	400<br>-	7.2	8.8	0.9	1.28	141	2	66
n.62 FSMP	183-205-218	-	-	-	-	-	-<br>-	-	-	-	-	-	-	-
n.63 FSMP	286	-	-	-	-	-	-<br>-	-	-	-	-	-	-	-
n.64 fortified food	-	-	-	-	-	-	-<br>-	-	1.6-1.8-2	-	-	-	-	-
n.65 FSMP	191	-	-	-	-	-	-<br>-	-	-	-	-	-	-	-
n.66 fortified food	22.4-29.4	1.9-20.4	-	6.3-6.4	-	-	163-169-175<br>-	-	43-44	-	-	-	-	86-116
n.67 FSMP	234	60	4.2	3.2	16.6	15	176	3.4	4.8	0.4	0.54	66	1.3	24

							β-carotene 0.058							
n.68 FSMP	125	34.1	2.38	1.8	9.5	8.5	100 β-carotene 0.032	1.91	2.75	0.22	0.31	37.5	0.74	13.6
n.69 FSMP	499.4	55	4.62	3.85	19.8	18.7	264 β-carotene 0.132	12.5	5.5	0.57	0.66	77	1.43	35.2
n.70 FSMP	300	80	4.2	4.8	18	20	316 -	3.4	6.8	0.6	0.68	96	1.28	40
n.71 FSMP	212.5	25	4.25	3.6	15	12.5	193.8 -	3.75	5.88	0.44	0.59	60	1.21	26.2
n.72 FSMP	200	60	4	3.6	19	16	300 -	2	3.8	0.42	0.6	60	1	36
n.73 FSMP	220	92.4	1.43	5.5	17.4	19.8	297 β-carotene 0.154	3.74	44	0.55	0.748	70.4	0.77	94.6
n.74 FSMP	187	39.6	2.86	2.42	13.2	11	220 -	1.39	3.96	0.37	0.57	55	0.88	19.8
n.75 FSMP	220	68.2	1.87	3.74	20.9	19.8	316.8 -	5.5	6.64	0.57	0.86	88	1.1	28.6
n.76	270.6	57.2	2.64	3.74	20.9	18.5	316.8	7	6.6	0.57	0.86	88	1.1	28.6

FSMP							-							
n.77 FSMP	25	-	-	-	-	-	-<br>-	-	-	-	-	-	-	-
n.78 FSMP	-	-	-	-	-	-	-<br>-	-	-	0.7	0.85	-	-	-
n.79 FSMP	-	-	-	-	-	-	-<br>-	-	-	-	-	-	-	-
n.80 supple ment	-	-	-	-	-	-	-<br>-	-	-	0.15	0.15	-	-	-
n.81 FSMP	-	-	-	-	-	-	-<br>-	-	-	-	-	-	-	-
n.82 FSMP	-	-	-	-	-	-	-<br>-	-	-	0.15	0.15	-	-	-
n.83 FSMP	-	-	-	-	-	-	-<br>-	10	-	0.15	0.15	-	-	-
n.84 FSMP	-	-	-	-	-	-	-<br>-	-	-	0.15	0.15	-	-	15
n.85 FSMP	-	-	-	-	-	-	-<br>-	-	-	0.15	0.15	-	-	15
n.86 FSMP	-	-	-	5	-	-	-<br>-	-	-	-	-	-	-	-

n.87 FSMP	-	-	-	5	-	-	- -	20	-	-	-	-	-	-
n.88 supplement	-	-	-	-	-	-	- -	-	-	-	-	-	-	-
n.89 supplement	-	-	-	-	-	-	- -	-	-	-	-	-	-	-
n.90 supplement	-	-	-	-	-	-	- -	-	-	-	-	-	-	-
n.91 supplement	33.8	≤ 0.5	-	-	-	-	- -	-	-	-	-	-	-	-
n.92 supplement	-	-	-	-	-	-	- -	-	-	-	-	-	-	-
n.93 FSMP	198	66	4.8	1.5	7.2	-	180 -	2.4	3.6	0.36	0.72	87	0.75	21
n.94 FSMP	66	22	1.6	0.5	2.4	-	60 -	0.8	1.2	0.12	0.24	29	0.25	7
n.95 FSMP	-	-	–	-	-	-	- -	-	-	-	-	-	-	-
n.96 FSMP	-	-	-	-	-	-	- -	-	-	-	-	-	-	-

n.97 FSMP	0.1	-	-	-	-	-	- -	-	-	-	-	-	-	-
n.98 FSMP	40.9	4.9	-	-	-	-	- -	10	-	-	1.7	-	-	-
n.99 FSMP	-	-	-	-	-	-	- -	-	-	0.15	0.15	-	-	-
n.100 FSMP	-	-	-	7.5	-	-	- -	10	-	0.25	0.2	-	-	35
n.101 FSMP	-	-	-	7.5	-	-	- -	10	-	0.25	0.2	-	-	35
n.102 FSMP	-	-	-	-	-	-	- -	-	-	-	-	-	-	-
n.103 FSMP	30	-	-	-	-	-	- -	-	-	-	-	-	-	-
n.104 FSMP	70	-	-	-	-	-	- -	-	-	-	-	-	-	-
n.105 FSMP	170	24	1.2	1.3	8.2	4.1	136 -	1.4	1.4	0.31	0.27	25.5	0.5	15.3
n.106 supplement	-	-	-	-	-	-	- -	-	-	-	-	-	-	-
n.107 FSMP	69.96	1.5	-	-	-	-	- -	-	-	-	-	-	-	-

n.108 supple ment	-	-	-	-	-	-	-<br>-	-	-	-	-	-	-	-
n.109 supple ment	-	-	-	-	-	-	-<br>-	-	-	-	-	-	-	-
n.110 supple ment	-	-	-	-	-	-	-<br>-	-	-	-	-	-	-	-
n.111 FSMP	215	-	-	-	-	-	-<br>-	-	-	-	-	-	-	-
n.112 FSMP	21.9	5.04	0.39	0.28	1.14	1.38	32.1<br>-	0.52	0.2	0.04	0.07	8.11	0.09	3.66
n.113 supple ment	321	31.4	1.31	1.4	12	12.5	256<br>β-carotene<br>0.011	5	4.3	0.43	0.55	71	0.81	29.4
n.114 FSMP	-	-	-	-	-	-	-<br>-	-	-	0.15	0.15	-	-	-
n.115 FSMP	-	-	-	-	-	-	-<br>-	-	-	0.3	0.3	-	-	-
n.116 FSMP	-	-	-	-	-	-	-<br>-	-	-	-	-	-	-	-
n.117 FSMP	-	-	-	-	-	-	-<br>-	-	-	-	-	-	-	-

n.118 FSMP	75	40	4	2	-	-	225<br>-	2	3	0.2	0.5	-	0.7	11
n.119 FSMP	-	-	-	-	-	-	-<br>-	-	-	0.22	0.28	-	-	-
n.120 FSMP	129	15	1	1	5	25.2	107.5<br>-	3	3.5	0.56	0.58	60	0.77	22.4
n.121 FSMP	-	-	-	-	-	-	-<br>-	-	-	-	-	-	-	-
n.122 FSMP	-	-	-	-	-	-	-<br>-	-	-	-	-	-	-	-
n.123 FSMP	-	-	-	-	-	-	-<br>-	-	-	0.07	0.1	-	-	-
n.124 FSMP	161	46.2	4.18	4.18	16.7	27.9	209<br>-	2.64	7.48	0.92	1.1	132	1.87	24.2
n.125 FSMP	233	46.2	4.18	4.18	16.3	9.9	209<br>-	2.64	7.48	0.92	1.1	132	1.87	23.1
n.126 FSMP	250	75	5	4.2	19.2	25	207.5<br>β-carotene<br>0.168	2.75	7.5	0.62	0.88	95	1.25	37.5
n.127 FSMP	7.5	< 0.75	4	3.4	20	16.2	0<br>carotenoid<br>s 0.56	1.75	6.25	0.375	0.75	125	0.59	7.5

n.128 FSMP	11.2	< 1.25	2.5	2.5	20	15	0 carotenoids 0.56	0	6.25	0.375	1.25	125	0.59	7.5
n.129 FSMP	193.8	18.8	4	3	28.8	16.2	158.8 carotenoids 0.75	1.75	6.25	0.375	0.44	66.2	0.625	50
n.130 FSMP	274	42	3.8	4	20	20	320 β-carotene 0.4	5	7	0.6	0.8	160	1.2	40
n.131 FSMP	490	130	8	8	45.5	45.5	585 β-carotene 0.65	6.5	10	1.3	1.3	195	1.95	65
n.132 FSMP	168	40	4	3.6	20	6	162.6 β-carotene 0.066	2	6	0.52	1.34	200	1.2	20

Supplementary Table S3. Cost analysis of the analyzed FSMPs and supplements. Abbreviations used: p: portion; b: brick; j: jar; s: sachet. Base prices are shown in the table, in the absence of discounts. Information on prices and indications for use were gathered from the companies' official websites. The monthly cost was calculated considering a month of 30 days. The Authors decided to not calculate month ly cost for complete nutrition, since the focus were FSMPs and supplements given in association to an habitual diet

Product n.	Cost per unit of sale	Cost per portion	Indications for use	Costs per day	Costs per month
n.1<br>FSMP	23€/can (280 g)	3.29 €/p (40g/p)	1-2 p/day with the diet	3.29-6.57€/day	98.70-197.14 €/month
n.2<br>FSMP	22€ for 4 bricks	5.50 €/b (200ml/b)	1-3 b/day with the diet	5.50-16.50 €/day	165-495 €/month
n.3<br>FSMP	22€ for 4 bricks	5.50 €/b (200ml/b)	1-3 b/day with the diet<br>5-7 b/day for complete nutrition	5.50-16.50 €/day<br>27.50-38.50 €/day	165-495 €/month<br>-
n.4<br>FSMP	19.5€ for 4 bricks	4.88 €/b (200ml/b)	1-3 b/day with the diet<br>5-7 b/day for complete nutrition	4.88-14.64 €/day<br>24.4-34.16 €/day	146.40-439.20 €/month<br>-
n.5<br>FSMP	20.5€ for 4 bricks	5.12 €/b (200ml/b)	1-3 b/day with the diet<br>7-10 b/day for complete nutrition	5.12-15.36 €/day<br>35.84-51.20 €/day	153.60-460.80 €/month<br>-
n.6<br>FSMP	19.5€ for 4 bricks	4.88 €/b (125ml/b)	1-3 b/day with the diet<br>5-7 b/day for complete nutrition	4.88-14.64 €/day<br>24.4-34.16 €/day	146.40-439.20 €/month<br>-
n.7<br>FSMP	19.5€ for 4 bricks	4.88 €/b (125ml/b)	1-3 b/day with the diet<br>5-7 b/day for complete nutrition	4.88-14.64 €/day<br>24.4-34.16 €/day	146.40-439.20 €/month<br>-
n.8<br>FSMP	20€ for 4 bricks	5 €/b (125ml/b)	1-3 b/day with the diet<br>5-7 b/day for complete nutrition	5-15 €/day<br>25-35 €/day	150-450 €/month<br>-
n.9<br>FSMP	16.5€ for 3 jars	5.5€/j (150 g/j)	1-3 j/day with the diet	5.50-16.50 €/day	165-495 €/month
n.10<br>FSMP	18.5€ for 4 bricks	4.62€/b (125ml/b)	1-2 b/day with the diet	4.62-9.24 €/day	138.60-277.20 €/month
n.11<br>FSMP	19.5€ for 4 bricks	4.88€/b (200ml/b)	1-3 b/day with the diet<br>5-7 b/day for complete nutrition	4.88-14.64 €/day<br>24.4-34.16 €/day	146.40-439.20 €/month<br>-

n.12 FSMP	30€ for 4 bricks	7.50€/b (200ml/b)	the dosage should be determined by the physician	-	-
n.13 FSMP	26€ for 4 bricks	6.5€/b (200 ml/b)	1-2 b/day with the diet	6.5-13 €/day	195-390 €/month
n.14 FSMP	-	- (200 ml/b)	2-3 b/day with the diet	-	-
n.15 FSMP	-	- (200 ml/b)	1-2 b/day with the diet 4-5 b/day for complete nutrition	-	-
n.16 FSMP	-	- (125 g/j)	3-4 j/day with the diet 7-8 j/day for complete nutrition	-	-
n.17 FSMP	-	- (125 ml/b)	1-2 b/day with the diet 4-5 b/day for complete nutrition	-	-
n.18 FSMP	-	- (120 ml/b)	3-4 b/day with the diet	-	-
n.19 FSMP	-	- (200 ml/b)	2-3 b/day with the diet 5 b/day for complete nutrition	-	-
n.20 FSMP	-	- (200 ml/b)	1-2 b/day with the diet	-	-
n.21 FSMP	-	- (125 ml/b)	2-3 b/day with the diet	-	-
n.22 FSMP	-	- (200 ml/b)	1-3 b/day with the diet 5-7 b/day for complete nutrition	-	-
n.23 FSMP	-	- (200 ml/b)	1-3 b/day with the diet 5-7 b/day for complete nutrition	-	-
n.24 FSMP	-	- (200 ml/b)	2-4 b/day with the diet 7-10 b/day for complete nutrition	-	-
n.25	-	- (200 ml/b)	1-3 b/day with the diet	-	-

FSMP			4-5 b/day for complete nutrition		
n.26<br>FSMP	-	- (200 ml/b)	1-3 b/day with the diet<br>5-7 b/day for complete nutrition	-	-
n.27<br>FSMP	-	- (200 ml/b)	2 b/day with the diet	-	-
n.28<br>FSMP	-	- (200 ml/b)	1-3 b/day with the diet	-	-
n.29<br>FSMP	-	- (200 ml/b)	1-2 b/day with the diet	-	-
n.30<br>FSMP	-	- (200 ml/b)	1-3 b/day with the diet	-	-
n.31<br>FSMP	-	- (200 ml/b)	1-3 b/day with the diet	-	-
n.32<br>FSMP	-	- (200 ml/b)	1-3 b/day with the diet	-	-
n.33<br>FSMP	-	- (125 g/j)	1-4j/day with diet	-	-
n.34<br>FSMP	-	- (125 g/j)	1-4j/day with diet	-	-
n.35<br>FSMP	-	- (125 g/j)	1-4j/day with diet	-	-
n.36<br>FSMP	59.91€ for 3 bricks	19.97€/b (237ml/b)	1-3b/day with diet	19.97-59.91 €/day	599.10-1797.30 €/month
n.37<br>FSMP	26.25€ for 4 bricks	6.56€/b (200ml/b)	1-2b/day with diet<br>3-4b/day for complete nutrition	6.56-13.12 €/day<br>19.68-26.24 €/day	196.80-393.60 €/month<br>-
n.38<br>FSMP	27.9€ for 4 bricks	6.98€/b (125ml/b)	1-3b/day with diet	6.98-20.94 €/day	209.40-628.20 €/month
n.39<br>FSMP	22.9€ for 4 bricks	5.72€/b (200ml/b)	1-2b/day with diet	5.72-11.44 €/day	171.60-343.20 €/month
n.40<br>FSMP	-	- (200ml/b)	1-3b/day with diet	-	-
n.41	27.90€ for 4 bricks	6.98€/b (125ml/b)	1-3b/day with diet	6.98-20.94 €/day	209.40-628.20 €/month

FSMP					
n.42<br>FSMP	19.90€ for 4 bricks	4.98€/b (200ml/b)	1-3b/day with diet	4.98-14.94 €/day	149.40-448.20 €/month
n.43<br>FSMP	22.9€ for 4 bricks	5.72€/b (200ml/b)	1-3b/day with diet<br>6-8b/day for complete nutrition	5.72-17.16 €/day<br>34.32-45.76 €/day	171.60-514.80 €/month<br>-
n.44<br>FSMP	23.6€ for 4 bricks	5.90€/b (200ml/b)	1-3b/day with diet<br>4-5b/day for complete nutrition	5.90-17.70 €/day<br>23.6-29.5 €/day	177-531 €/month<br>-
n.45<br>FSMP	34.9€ for 4 bricks	8.72€/b (200ml/b)	1-3b/day with diet	8.72-26.16 €/day	261.60-784.80 €/month
n.46<br>FSMP	27.9€ for 4 bricks	6.98€/b (200ml/b)	1-3b/day with diet<br>6-8b/day for complete nutrition	6.98-20.94 €/day<br>41.88-55.84 €/day	209.40-628.20 €/month<br>-
n.47<br>FSMP	17.96€ for 4 bricks	4.49€/b (200ml/b)	1-3b/day with diet	4.49-13.47 €/day	134.70-404.10 €/month
n.48<br>supplement	19.99€/can (270 g)	13.32€/p (30g/p)	1-2p/day with diet	13.32-26.65 €/day	399.60-799.60 €/month
n.49<br>fortified food	21.99€ for 3 jars	7.33€/j (125g/j)	1-3j/day with diet	7.33-21.99 €/day	219.90-659.70 €/month
n.50<br>FSMP	4.49€ for 1 brick	4.49€/b (200g/b)	1-2b/day with diet	4.49-8.98 €/day	134.70-269.40 €/month
n.51 fortified food	14.90 € for 4 jars	3.72€/j (100g/j)	1-3j/day with diet	3.72-11.16 €/day	111.60-334.80 €/month
n.52<br>FSMP	5.49€ for 1 brick	5.49€/b (200ml/b)	1-3b/day with diet	5.49-16.47 €/day	164.70-494.10 €/month
n.53<br>FSMP	23.90€ for 3 jars	7.97€/j (125ml/j)	1-3j/day with diet<br>6-8j/day for complete nutrition	7.97-23.91 €/day<br>47.82-63.76 €/day	239.10-717.30 €/month<br>-
n.54<br>FSMP	5.40€ for 1 brick	5.40€/b (200ml/b)	2b/day with diet<br>the dosage for complete nutrition should be determined by the physician	10.80 €/day<br>-	324 €/month<br>-
n.55<br>FSMP	6.30€ for 1 brick	6.30€/b (200ml/b)	2b/day with diet	12.60 €/day<br>-	378 €/month<br>-

			the dosage for complete nutrition should be determined by the physician		
n.56<br>FSMP	3.30€ for 1 brick	3.30€/b (200ml/b)	2b/day with diet<br>the dosage for complete nutrition should be determined by the physician	6.60 €/day<br>-	198 €/month<br>-
n.57<br>FSMP	3.70€ for 1 brick	3.70€/b (200ml/b)	2b/day with diet<br>the dosage for complete nutrition should be determined by the physician	7.40 €/day<br>-	222 €/month<br>-
n.58<br>FSMP	27.60€ for 4 bricks	6.90€/b (200ml/b)	2b/day with diet<br>the dosage for complete nutrition should be determined by the physician	13.80 €/day<br>-	414 €/month<br>-
n.59<br>FSMP	11€ for 1 bricks	11€/b (500ml/b)	1-3b/day with diet	11-33 €/day	330-990 €/month
n.60<br>FSMP	10.60 €<br>for 1 brick	10.60€/b (500ml/b)	2b/day with diet<br>the dosage for complete nutrition should be determined by the physician	21.20 €/day<br>-	636 €/month<br>-
n.61<br>FSMP	12.10 € for 1 brick	12.10€/b (200 ml/b)	2b/day with diet<br>the dosage for complete nutrition should be determined by the physician	24.20 €/day<br>-	726 €/month<br>-
n.62<br>FSMP	5.5 € for 1 jar	5.5€/j (125 g/j)	2-3j/day with diet	11-16.50 €/day	330-495 €/month
n.63<br>FSMP	6 € for 1 jar	6 €/j (125g/j)	2-3j/day with diet	12-18 €/day	360-540 €/month

n.64<br>fortified food	2.42 €<br>for 1 cup	2.42 €/j (125g/j)	1-3j/day with diet	2.42-7.26 €/day	72.60-217.80 €/month
n.65<br>FSMP	20.70 € for 4 jars	6.9 €/j (125 g/j)	2-3j/day with diet	13.80-20.70 €/day	414-621 €/month
n.66<br>fortified food	6.40 € for 1 jar	6.4€/j (125 g/j)	2-3j/day with diet	12.80-19.20 €/day	384-576 €/month
n.67<br>FSMP	-	- (200 ml/b)	1-2b/day with diet	-	-
n.68<br>FSMP	-	- (125 g/j)	-	-	-
n.69<br>FSMP	-	- (220 ml/b)	2b/day with diet	-	-
n.70<br>FSMP	-	- (200 ml/b)	1-2b/day with diet	-	-
n.71<br>FSMP	-	- (125 ml/b)	1-2b/day with diet	-	-
n.72<br>FSMP	-	- (200 ml/b)	1-2b/day with diet	-	-
n.73<br>FSMP	-	- (220 ml/b)	2b/day with diet	-	-
n.74<br>FSMP	-	- (220 ml/b)	1-2b/day with diet	-	-
n.75<br>FSMP	-	- (220 ml/b)	-	-	-
n.76<br>FSMP	-	- (220 ml/b)	-	-	-
n.77<br>FSMP	-	- (5 g)	1-2s/day with diet	-	-
n.78<br>FSMP	-	- (5.85 g)	1-2s/day with diet	-	-
n.79<br>FSMP	-	- (-)	-	-	-
n.80<br>supplement	-	- (5.5 g)	1-2s/day with diet	-	-
n.81	-	- (5.5 g)	1-2s/day with diet	-	-

FSMP					
n.82<br>FSMP	-	- (7 g)	2s/day with diet	-	-
n.83<br>FSMP	-	- (6.5 g)	1-2s/day with diet	-	-
n.84<br>FSMP	-	- (25 g)	2s/day with diet	-	-
n.85<br>FSMP	-	- (25 g)	2s/day with diet	-	-
n.86<br>FSMP	-	- (5.5 g)	1-2s/day with diet	-	-
n.87<br>FSMP	-	- (5.5 g)	1-2s/day with diet	-	-
n.88<br>supplement	-	- (5 g)	the dosage should be determined by the physician	-	-
n.89<br>supplement	- (200g/can)	- (5 g)	the dosage should be determined by the physician	-	-
n.90<br>supplement	- (400g/can)	- (5 g)	the dosage should be determined by the physician	-	-
n.91<br>supplement	- (225g/can)	- (2.5 g)	the dosage should be determined by the physician	-	-
n.92<br>supplement	-	- (5.7 g)	1s/day with diet	-	-
n.93<br>FSMP	- (400g/can)	- (30 g)	1p/day with diet	-	-
n.94<br>FSMP	-	- (10 g)	1p/day with diet	-	-
n.95<br>FSMP	- (250g/can or 500g/can)	- (10 g)	1p/day with diet	-	-

n.96<br>FSMP	- (325g/can)	- (20 g)	the dosage should be determined by the physician	-	-
n.97<br>FSMP	- (500g/can)	- (10 g)	the dosage should be determined by the physician	-	-
n.98<br>FSMP	-	- (21 g)	1s/day with diet	-	-
n.99<br>FSMP	-	- (5.5 g)	1-2s/day with diet	-	-
n.100<br>FSMP	-	- (5.5 g)	1-2s/day with diet	-	-
n.101<br>FSMP	-	- (10 g)	1-2s/day with diet	-	-
n.102<br>FSMP	-	- (5 g)	the dosage should be determined by the physician	-	-
n.103<br>FSMP	69.90€ (300 g/can)	3.50€/p (15 g/p)	1-3p/day with diet	3.50-10.50 €/day	105-315 €/month
n.104<br>FSMP	59.90€ (400 g/can)	2.24€/p (15 g/p)	1-3p/day with diet<br>the dosage should be determined by the physician	2.24-6.72€/day	67.20-201.60 €/month
n.105<br>FSMP	19.99€ (270 g/can)	2.52 €/p (34 g/p)	1-3p/day with diet	2.52-7.55 €/day	75.60-226.50 €/month
n.106<br>supplement	44€ (500 g/can)	1.06€/p (12g/p)	the dosage should be determined by the physician	-	-
n.107<br>FSMP	75€ (550 g/can)	1.63€/p (12 g/p)	the dosage should be determined by the physician	-	-
n.108<br>supplement	50.01€ (500 g/can)	0.50€/p (5 g/p)	the dosage should be determined by the physician	-	-

n.109 supplement	24.90€ (600 g/can)	0.29€/p (7 g/p)	the dosage should be determined by the physician	-	-
n.110 supplement	60€ for 24 sachets	2.5€/s (5 g/s)	the dosage should be determined by the physician	-	-
n.111 FSMP	-	- (24 g/s for orange; 19.3 g/s for neutral flavour)	2s/day with diet	-	-
n.112 FSMP	-	- (76 g/s)	-	-	-
n.113 supplement	- (400g/can or 800g/can)	- (53.5 g/p)	1-2p/day with diet	-	-
n.114 FSMP	36€ for 20 sachets	1.8€/s (6.3 g/s)	2s/day with diet	3.6 €/day	108 €/month
n.115 FSMP	36€ for 20 sachets	1.8€/s (6.5 g/s)	2-3s/day with diet	3.6-5.4 €/day	108-162 €/month
n.116 FSMP	48 €/can (400 g)	0.56/p (6g/p)	the dosage should be determined by the physician	-	-
n.117 FSMP	45 €/can (300 g)	1.77€/s (10g/s)	the dosage should be determined by the physician	-	-
n.118 FSMP	14.80 €/can (250g)	1.74€/p (12.5 g/p)	1-2p/day with diet	1.74-3.48 €/day	52.20-104.40€/month
n.119 FSMP	96 € for 30 sachets	3.2€/s (25g/s)	1s/day with diet	3.2€/day	96 €/month
n.120 FSMP	-	- (50 g)	1s/day with diet	-	-
n.121 FSMP	35.91 € for 20 sachets	1.8€/s (5g/s)	2-3s/day with diet	3.6-5.4 €/day	108-162 €/month
n.122 FSMP	59 €/can (300g)	2.95€/s (15g/p)	2p/day with diet	5.9 €/day	177 €/month
n.123 FSMP	15 €/can (500g)	0.45€/p (15 g/p)	the dosage should be determined by the physician	-	-

n.124<br>FSMP	-	- (220 ml/b)	1-2 b/day with diet or for complete nutrition	-	-
n.125<br>FSMP	-	- (220 ml/b)	1-2 b/day with diet or for complete nutrition	-	-
n.126<br>FSMP	-	- (250 ml/b)	-	-	-
n.127<br>FSMP	-	- (125 ml/b)	1-3 b/day with diet	-	-
n.128<br>FSMP	-	- (125 ml/b)	1-3 b/day with diet	-	-
n.129<br>FSMP	-	- (125 ml/b)	3 b/day with diet	-	-
n.130<br>FSMP	-	- (200 ml/b)	1-3 b/day with diet<br>4-5 b/day for complete nutrition	-	-
n.131<br>FSMP	-	- (500 ml/b)	3-4 b/day for complete nutrition	-	-
n.132<br>FSMP	-	- (200 ml/b)	2-3 b/day with diet<br>≥ 5 b/day for complete nutrition	-	-
